# Influence of Intramineral Proteins on the Growth of Carbonate Crystals Using as a Scaffold Membranes of Ratite Birds and Crocodiles Eggshells

**DOI:** 10.3390/membranes13110869

**Published:** 2023-11-01

**Authors:** Nerith R. Elejalde-Cadena, Denisse Hernández, Francesco Capitelli, Selene R. Islas, Maria J. Rosales-Hoz, Michele Zema, Serena C. Tarantino, Dritan Siliqi, Abel Moreno

**Affiliations:** 1 Institute of Physics, National Autonomous University of Mexico, Circuito de la Investigación Científica s/n, Ciudad Universitaria, Ciudad de Mexico 045010, Mexico; rocioec@fisica.unam.mx; 2 Institute of Chemistry, National Autonomous University of Mexico, Av. Universidad 3000, Ciudad de Mexico 04510, Mexico; 316298290@quimica.unam.mx; 3 Institute of Crystallography (IC), National Research Council (CNR), Via Salaria km 29,300, 00016 Rome, Italy; francesco.capitelli@ic.cnr.it; 4 Instituto de Ciencias Aplicadas y Tecnología, Universidad Nacional Autónoma de México, Circuito Exterior s/n, Cd. Universitaria, Ciudad de Mexico 045010, Mexico; selene.islas@icat.unam.mx; 5 Departamento de Química, Centro de Investigación y de Estudios Avanzados, Av. Instituto Politécnico Nacional 2508, Col. San Pedro Zacatenco, Ciudad de Mexico 07360, Mexico; mrosales@cinvestav.mx; 6 Department of Earth and Geoenvironmental Sciences, University of Bari “Aldo Moro”, Via E. Orabona 4, 70125 Bari, Italy; michele.zema@uniba.it; 7 Institute of Crystallography (IC), National Research Council (CNR), Via Amendola 122/O, 70126 Bari, Italy; 8 Department of Chemistry, University of Pavia, Vialle Taramelli 16, 27100 Pavia, Italy; serenachiara.tarantino@unipv.it; 9 Institute of Geoscience and Georesources (IGG), National Research Council (CNR), Via Ferrata 1, 27100 Pavia, Italy

**Keywords:** membrane, eggshell, biomineralization, intramineral proteins, biocalcification, biosilicification, biomorphs

## Abstract

The lack of information on structural basis where proteins are involved, as well as the biomineralization processes of different systems such as bones, diatom frustules, and eggshells, have intrigued scientists from different fields for decades. This scientific curiosity has led to the use of methodologies that help understand the mechanism involved in the formation of these complex structures. Therefore, this work focuses on the use of eggshell membranes from different species of ratites (emu and ostrich) and reptiles (two species of crocodiles) as a model to differentiate biocalcification and biosilicification by introducing calcium phosphate or silica inside the membrane fiber mantles. We performed this to obtain information about the process of eggshell formation as well as the changes that occur in the membrane during crystal formation. In order to identify and understand the early processes leading to the formation of the microstructures present in the eggshell, we decided to carry out the synthesis of silica-carbonate of calcium, barium, and strontium called biomorph in the presence of intramineral proteins. This was carried out to evaluate the influence of these proteins on the formation of specific structures. We found that the proteins on untreated membranes, present a structural growth similar to those observed in the inner part of the eggshell, while in treated membranes, the structures formed present a high similarity with those observed in the outer and intermediate part of the eggshell. Finally, a topographic and molecular analysis of the biomorphs and membranes was performed by scanning electron microscopy (SEM), Raman and Fourier-transform Infrared (FTIR) spectroscopies.

## 1. Introduction

Biomineralization is the process by which living organisms can produce inorganic materials (carbonates, phosphates, oxalates, sulfates, oxides, and silicates) [1,2]. According to the degree of biological control, two types of biomineralization are known: (1) Biologically induced biomineralization that occurs by interacting with the environment. This is a biologically induced process where the size, shape, morphology, and organization of the structures are indefinite and heterogeneous, resulting in the pathological mineralization observed in kidney stones and/or gallstones [3,4,5]; (2) Biomineralization induced by an organic matrix that allows highly controlled mineralization by biological macromolecules such as proteins, polysaccharides, and lipids located in a macromolecular network that allows the formation of bones, teeth, mollusk shells, and eggshells, among others [6,7].

Little is known about the structural information of the macromolecules and genes that constitute and act in living organisms during these processes. This is due to the level of organization of biominerals which is often hierarchical in different structural orders, to produce a system with a unique morphology and properties that have not yet been reproduced by man [8]. Among the processes mediated by a membrane, three groups stand out in nature where the importance of biological entities can be appreciated. The first are diatoms. Diatoms have a rigid and porous cell wall called a frustule which is composed of amorphous SiO_2_ silica. The second are the structures composed of calcium phosphates, on all Ca_10_(PO_4_)_6_(OH)_2_ hydroxyapatite, as we have seen in bones, teeth, shrimp shells, etc. We finally have one equally important class: the CaCO_3_ calcium carbonate compounds, found mainly in mollusks and eggshells. However, the percentage of silica, phosphate or carbonate may vary depending on the species, and/or in the case of calcium carbonate, on polymorphs, such as vaterite, aragonite or where calcite may be present [9,10,11].

It is known that eggshells have an organic phase consisting of sugars, lipids, and proteins, and an inorganic phase composed mainly of calcium carbonate in calcite form corresponding to the eggshell’s mineral part. The mineral part is constituted by the mammillary knobs, which are conical calcium carbonate crystals that give way to the formation of the palisade layer made up of columns to allow the formation of pores. This is followed by the formation of a vertical crystal layer that separates the palisade layer from the cuticle, which is located on the outside of the eggshell and allows the coloration of the eggshell to be seen after oviposition [12].

However, the biomineralization of eggshells is mainly controlled by the proteins that compose the organic matrix. This process occurs in the uterus, a cellular membrane rich in minerals and proteins necessary for eggshell formation. More than 50 proteins have been identified in the uterine fluid that performs different functions, such as the formation of proteoglycans, chaperone, proteases, antibacterial, and intramineral proteins [13,14].

Intramineral proteins are the ones that play a major role in the biomineralization process and are mainly found in the mineral phase of the eggshell. The first intramineral protein to be structurally characterized was ovocleidin-17 (OC-17), isolated from chicken eggshell [15,16,17]. Proteins homologous to OC-17 have also been found in eggshells of other species such as the protein ansocalcin (ANCA) that has been isolated from goose [18]. Struhiocalcins-1 and -2 (SCA-1 and SCA-2), dromaiocalcins-1 and -2 (DCA-1 and DCA-2), and rheacalcins-1 and -2 (RCA-1 and RCA-2) have been isolated from ratites such as ostrich, emu, and rhea [19,20], respectively.

So far, most of the investigations have been focused on the process of eggshell formation in hens, describing all steps of egg formation. However, there is still a lack of structural information on intramineral proteins related to *Neognathae*, *Paleognathae,* and *Crocodylidae* species. Consequently, the aim of this work is to realize the growth of biomorphs, which are silica-carbonate structures that mimic the complex and highly ordered structures formed in nature by biomineralization processes [21,22]. Therefore, the growth of biomorphs of different alkaline earth elements, such as calcium (Ca), barium (Ba), and strontium (Sr), in presence of intramineral proteins isolated from the respective eggshells of ostrich, emu, and two species of crocodiles will help us to understand the influence of intramineral proteins in the growth of calcium carbonate crystals. The most interesting aspect of this is that it will provide information about the process of formation, nucleation, and growth of the mineral phase (calcite crystals) of the eggshells of ratite birds and archosaurian reptile, species which are phylogenetically related to chickens.

## 2. Materials and Methods

### 2.1. Eggshells Cleaning and Membranes Separation

Eggshells of ostrich (*Struthio camelus*), emu (*Dromaius novahollandiae*), marsh crocodile (*Crocodylus moreletti*), and river crocodile (*Crocodylus acutus*) were washed with a 5% EDTA (ethylenediaminetetraacetic acid) solution for 45 min at room temperature. The membrane was then removed from the eggshell and washed with milli-Q water to remove excess EDTA and finally stored in water at 4 °C for further analysis.

### 2.2. Intramineral Proteins Isolation

Once the eggshells were clean and dry, they were crushed to obtain a fine powder, which was dissolved in 10% acetic acid (AcOH) at a ratio of 20 mL of AcOH per 1 g of eggshell and kept under agitation for 72 h at 4 °C. The samples were then placed at a temperature of −30 °C for 12 h to perform the cryo-concentration process, following the steps of Virgen-Ortiz et al. [23]. Finally, the solution was concentrated for 30 min, at 6000 rpm, at 4 °C, using a LISA centrifuge AFI-C200R and a 3 kDa and 10 kDa molecular cutting membrane, respectively. The concentrate was separated for further protein purification.

### 2.3. Purification and Characterization of Intramineral Proteins

Protein separation and purification was performed by Fast Protein Liquid Chromatography (FPLC) on an AKTA PURE using a Superdex 75 Increase 10/300 GL gel filtration column and a sample volume of 0.5 mL. The flow rate was 0.5 mL/min at 1.5 CV (Column volume) using 50 mM sodium citrate + 150 mM NaCl pH 4.0 as mobile phase.

Protein identification was performed by denaturing electrophoresis in SDS-PAGE (sodium dodecyl sulfate-polyacrylamide gel electrophoresis), by determining characteristic bands or patterns of the proteins obtained. The gels were run on a Mini-PROTEAN 3 vertical separation system (Biorad, Hercules, CA, USA), with glass plates 0.75 mm thick, 10 cm high, and 7 cm wide. Samples were incubated with the loading buffer in a 2:1 sample; buffer ratio for 5 min at 90 °C and a loading intensity of 30 V was used for 30 min (until the bands were on the separating gel), then 120 V for 1 h 45 min until the end of the run on the gel. The gels were stained with Coomasie blue R-250 at 0.5% *w/v* in AcOH:Methanol:H_2_O (1:4:5) at room temperature for 1 h, then destained with a destaining solution for 30 min. Finally, the gels were left in milli-Q water for 12 h.

### 2.4. Determination of Molecular Weight of Isolated Intramineral Proteins

The molecular weight of the proteins was determined by MALDI-TOF (matrix-assisted laser desorption/ionization) mass spectrometry using a Buker Esquire spectrometer with a matrix of sinapinico acid (SA) in a 1:5 matrix:protein ratio, which allows ionization of the matrix and protein by laser irradiation. Once the molecules are ionized, separation is performed according to the mass-to-charge ratio.

### 2.5. Synthesis of Biomorphs

Biomorphs were obtained using chlorides of alkaline earth elements such as calcium (Ca), barium (Ba), and strontium (Sr) by the gas diffusion method [24]. The synthesis was carried out using 5 × 5 cm glasses. The glasses were placed in a reservoir inside a crystallization chamber containing 100 µL of a 20 mM Ca^2+^, Ba^2+^, and Sr^2+^ chloride solution and 100 µL of 4500 ppm sodium metasilicate, adjusted pH 11 with 5 µL of 500 mM NaOH. They were incubated at room temperature for 24 h and washed with milli-Q water, ethanol, and air dried.

### 2.6. Morphological Characterization by Scanning Electron Microscopy (SEM)

Analyses were performed using a SEM-TESCAN model VEGA3 SB microscope with a voltage of 10 keV in high vacuum using backscattered electrons (BSE). All samples were coated with gold for 30 s to improve conductivity. The samples were placed on aluminum stubs and held in place with carbon tape.

### 2.7. Vibrational Spectrocopy Characterization of Biomorphs

Raman spectra were collected using an alpha300 RA spectrometer (WITec GmbH, Ulm, Germany) under ambient conditions with 532 nm laser light excitation, from a Nd:YVO_4_ incident laser beam, with a power of 31.7 mW and detection of 300 lines/mm grating. The incident laser beam was focused by 50× and 100× objectives (Oberkochen, Germany) with 0.75 and 0.9 NA, respectively.

Punctual Raman spectra were obtained with 0.5 s of integration time and 0.03 s for imagen mapping. The data processing and analysis were performed with the WITec Project Version 5.1 software.

Fourier-transform Infrared (FTIR) analysis was performed on a Nicolet iS50R Thermo-Scientific (Waltham, MA, USA) spectrometer using the attenuated total reflectance (ATR) method with a diamond crystal accessory (Smart-iTX). Spectra were acquired with 32 scans, 4 cm^−1^ of resolution in the range of 525 to 4000 cm^−1^.

### 2.8. Elemental Analysis of Membranes by Scanning Electron Microscopy and Energy Dispersive X-ray Spectroscopy (SEM-EDS)

Elemental analysis of the membranes was performed using a JEOL JSM-7800F microscope (Peabody, MA, USA) with variable magnification at 15 keV. The procedure was carried out with 150 s. The membranes were placed on an aluminum stub, fixed with carbon tape, and coated with gold as described in Section 2.6.

### 2.9. Biocalcification and Biosilicification of Membranes

In order to understand the biocalcification process, the membranes were treated with a 1.25 N 3-mercaptopropionic acid solution in 10% acetic acid for 3 h according to the methodology of Li et al. [25]. Subsequently, they were washed with milli-Q water and immersed in a 5% sodium triphosphate solution for 1 h at room temperature. After this time, the membranes were washed with milli-Q water and transferred to a calcium phosphate solution (10.5 mM CaCl_2_ + 6.3 mM K_2_HPO_4_) in HEPES pH 7.4. Spontaneous precipitation of hydroxyapatite was prevented by the addition of 500 µg/mL polyacrylic acid. Incubation was performed at 37 °C for 28 days with daily solution changes.

For biosilicification, orthosilicic acid oligomers were prepared by stirring 40% hydrolyzed tetraethyl orthosilicate in a molar ratio of 1:211.62:6.42 of absolute ethanol:H_2_O: 37% HCl for 1 h at room temperature. The previously treated membranes with 3-mercaptopropionic acid, were immersed in the solution containing the oligomers for 1 h and washed with milli-Q water. A 3% silicic acid solution was then prepared with 0.07 M choline chloride and stirred for 1 h. After this time, the solution was centrifuged at 3000 rpm and the supernatant containing the choline-stabilized silicic acid was collected. The membranes were placed in 1 mL of the choline-stabilized solution at 37 °C for 4 days, with daily solution changes.

### 2.10. Production of Biomorphs Using Eggshell Membranes as Scaffolds

Biomorphs were obtained using the same method as above, with a protein concentration of 0.1 mg/mL and using 5 × 5 mm membrane sections instead of glass, which were inserted into the reservoirs and incubated for 24 h at room temperature.

## 3. Results and Discussions

### 3.1. Separation of the Organic Membrane Present in the Eggshells

Eggshells from ostrich, emu, and the two crocodilian species were washed in a 5% EDTA solution for 45 min to separate the organic membrane and remove impurities present in the samples. The membranes were then separated to prevent rupture, washed with milli-Q water, and stored in water at 4 °C to avoid dehydration of the membranes.

### 3.2. Extraction and Characterization of the Intramineral Proteins

Approximately 50 g of ostrich and emu eggshells and 3 g of crocodile eggshells were crushed. These were dissolved in 1 L (for birds) and 40 mL (for reptiles) of 10% AcOH, which was added gradually to avoid uncontrolled foaming due to the formation of gaseous CO_2_, which would cause the loss of the sample. The solution containing the eggshells was stirred at 4 °C for 72 h until no effervescence was observed. Subsequently, the extract was filtered twice using a Büchner funnel-type system with a Millipore filter of 0.22 μm and concentrated using the cryo-concentration technique based on the use of the colligative properties of the water–protein system [23], allowing to obtain the protein due to its low freezing point, which was dialyzed by using a 3 kDa cut-off membrane in 5% AcOH for 24 h under constant agitation at 4 °C, to remove low molecular weight particles and restore equilibrium. Finally, the extract was concentrated using Millipore tubes with a 10 kDa membrane filter at 3500 rpm for 15 min at 4 °C, giving a final concentration of 2.2 mg/mL in 5.0 mL of extract for ostrich. For emu, a final concentration of 2.5 mg/mL was obtained in a volume of 4.0 mL, 1.6 mg/mL, and 1.8 mg/mL in a final volume of 3.0 and 3.5 mL for the crocodile *acutus* and *moreletti*, respectively.

**Figure 1:**
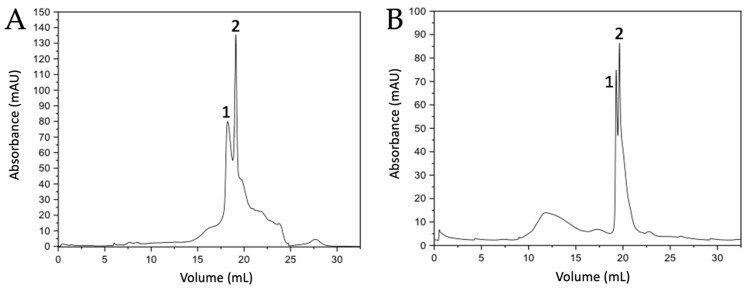
Chromatograms of the separation of intramineral proteins from ostrich (**A**) and emu (**B**) eggshells obtained by gel filtration method. Numbers 1 and 2 indicate the peaks collected for further analysis.

Protein separation was performed by molecular exclusion chromatography on FPLC, from which 0.5 mL fractions were collected for each of the samples. It should be noted that the protein purification procedure was optimized by performing several tests with different buffers and pH. From the chromatogram obtained for ostrich (Figure 1A), six fractions were collected (Table S1), which were observed by SDS-PAGE gel (Figure S1A), revealing bands with a molecular weight of ~17 kDa and ~15 kDa, respectively. In the case of emu eggshell, similar results were observed with a collection of six fractions (Table S1), which presented bands of molecular weight close to ~16 kDa and ~15 kDa (Figure S1B).

**Figure 2:**
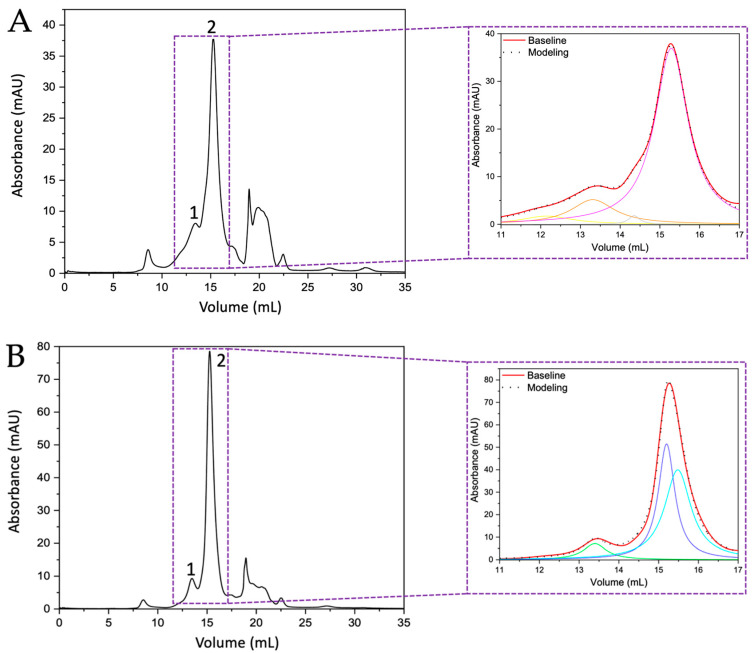
Chromatograms of the separation of intramineral proteins from crocodile *acutus* (**A**) and *moreletti* (**B**) eggshells obtained by gel filtration method. Numbers 1 and 2 indicate the peaks collected for further analysis. The inset shows a magnification and deconvolution of the area where intramineral proteins are found. Orange: ~14 kDa protein of crocodile *acutus*; Pink: ~7 kDa protein of crocodile *acutus*; Green: ~14 kDa protein of crocodile *moreletti*; Blue and Aguamarine: ~7 kDa proteins of crocodile *acutus*.

For the purification of intramineral proteins from the eggshell of crocodile *acutus* and *moreletti*, the gel filtration method implemented for the separation of ostrich and emu proteins was used. In the case of crocodile *acutus* (Figure 2A), eight fractions were collected (Table S2), where fractions three and four had very weak bands with a molecular weight of ~14 kDa, while fractions five to seven had bands characteristic of a protein of ~7 kDa (Figure S2A). Something similar was observed in the crocodile *moreletti* eggshell (Figure 2B), where seven fractions were collected. In this case, a very faint band at ~14 kDa was observed in fraction three and the fractions five to seven presented an intense band at about ~7 kDa (Figure S2B).

By comparing the chromatograms obtained from the ratite birds with those of the crocodile species, it can be observed that the widths of peak 2 of the crocodile chromatograms (Figure 2A,B) are similar to those observed for the two peaks in the chromatograms of the ratite birds (Figure 1A,B). Therefore, the deconvolution of those two peaks observed in the chromatograms obtained from the crocodile eggshells was performed by MagicPlot 2.9.3 Software, where Lorentzian functions were adjusted to identify the presence of other proteins and it is evident that in the crocodile *acutus* eggshell there are two curves corresponding to two proteins, one of ~7 kDa (Figure 2A inset, peak 2, pink line) and the other of ~14 kDa (Figure 2A inset, peak 1, orange line); the peak in green and yellow correspond to impurities confirmed by SDS-PAGE and Maldi-ToF. However, in the case of the crocodile *moreletti* eggshell, deconvolution revealed the presence of two ~7 kDa proteins (Figure 2B inset, peak 2, blue and aquamarine lines) and one ~14 kDa protein (Figure 2B inset, peak 1, green line).

Once the intramineral proteins were identified, the characteristic protein signals were analyzed by MALDI-TOF mass spectrometry. In the case of ratite birds, peaks 1 and 2 of the ostrich chromatogram (Figure 1A) showed molecular weights of 15,353 Da and 16,755 Da (Figure S3A,B), corresponding to the proteins struthiocalcin-1 and -2 (SCA-1 and SCA-2). In the case of emu (Figure 1B), the isolated peaks presented molecular weights of 15,779 and 16,837 Da (Figure S3C,D), named dromaiocalcin-1 and -2 (DCA-1 and DCA-2), respectively [26,27]. In the chromatograms (Figures S3 and S4), it is observed that there is an additional peak in the mass spectra, where the largest peak corresponds to the molecular ion (M^1+^) and the smallest to the doubly charged species. This is because the charge of the proteins causes the double charge species to have a molecular weight half that of the singly charged molecular ion [28,29]. In the case of reptiles, the proteins isolated from the eggshells of crocodile *acutus* had a molecular weight of 14,139 and 7040 Da (Figure S4A,B). In a sense and following the designation of Legorreta et al. [30], these proteins have been named crococalcins. However, the proteins isolated from crocodile *acutus* will be referred to as CCA-7 and CCA-14, for the 7 and 14 kDa proteins, in order to avoid confusion of the assigned acronyms. From crocodile *moreletti*, peak one obtained a molecular weight of 14,075 Da (Figure S4E) and peak two, from which two proteins were isolated, presented a molecular weight of 7034 and 7036 Da (Figure S4C,D). These proteins were named CCM-1, CCM-2, and CCM-3, indicating that they are crococalcins (CC) from crocodile *moreletti* (M).

### 3.3. Formation and Determination of the Molecules Present in the Synthethized Biomorphs

**Figure 3:**
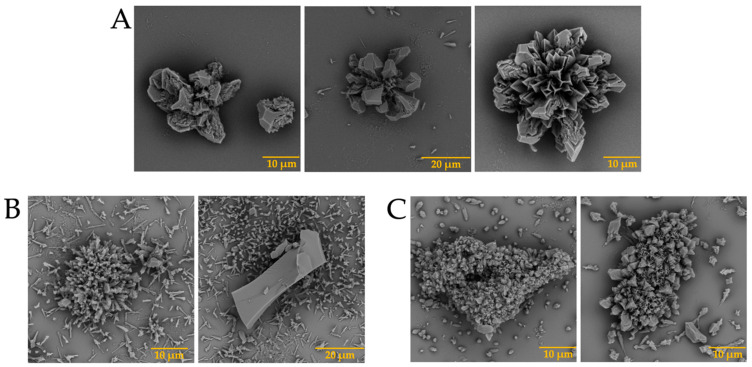

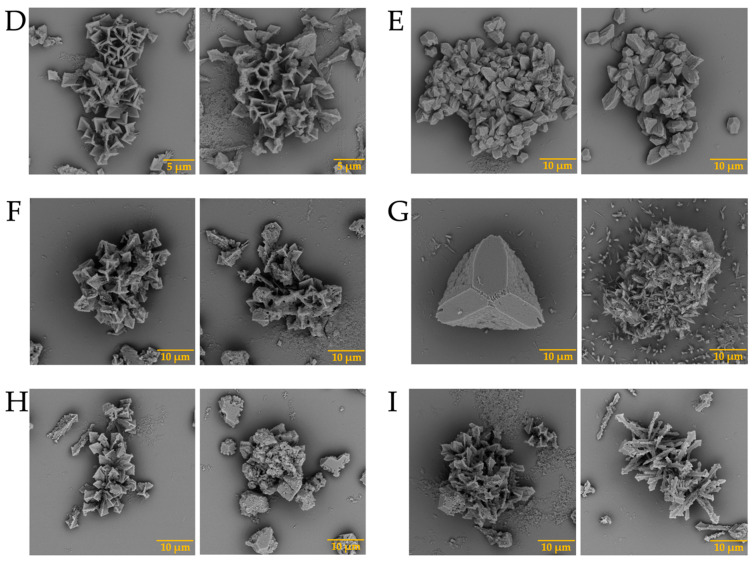
SEM micrographs of calcium silica-carbonates structures in the presence of intramineral proteins. (**A**) Control; (**B**) SCA-1; (**C**) SCA-2; (**D**) DCA-1; (**E**) DCA-2; (**F**) CCA-7; (**G**) CCA-14; (**H**) CCM-1; (**I**) CCM-3.

The presence of biomolecules is known to be necessary for the formation of well-defined structures [31]. Although science has evolved over the years, the mechanism of eggshell formation is not yet fully understood [32,33]. This is why we decided to carry out the test with calcium, barium, and strontium silica-carbonate biomorphs in the presence of the previously isolated intramineral proteins. The first test was the formation of calcium biomorphs in the presence of SCA, DCA, CCA, and CCM proteins. According to Figure 3A, the control structures of calcium biomorphs have a drusy morphology, while in the presence of intramineral proteins, polycrystalline, granular and flower morphologies with spicules were obtained (Figure 3B–I). In contrast to the biomorphs synthesized in the presence of CCA-14 (Figure 3G), pyramidal and acicular structures were observed. However, the morphologies in the structures with proteins did not show a significant change compared to those obtained without the influence of proteins. We, therefore, decided to perform a phase qualitative investigation of each of the tests by Raman spectroscopy, due to the reliability of the results obtained by this technique [34].

**Figure 4:**
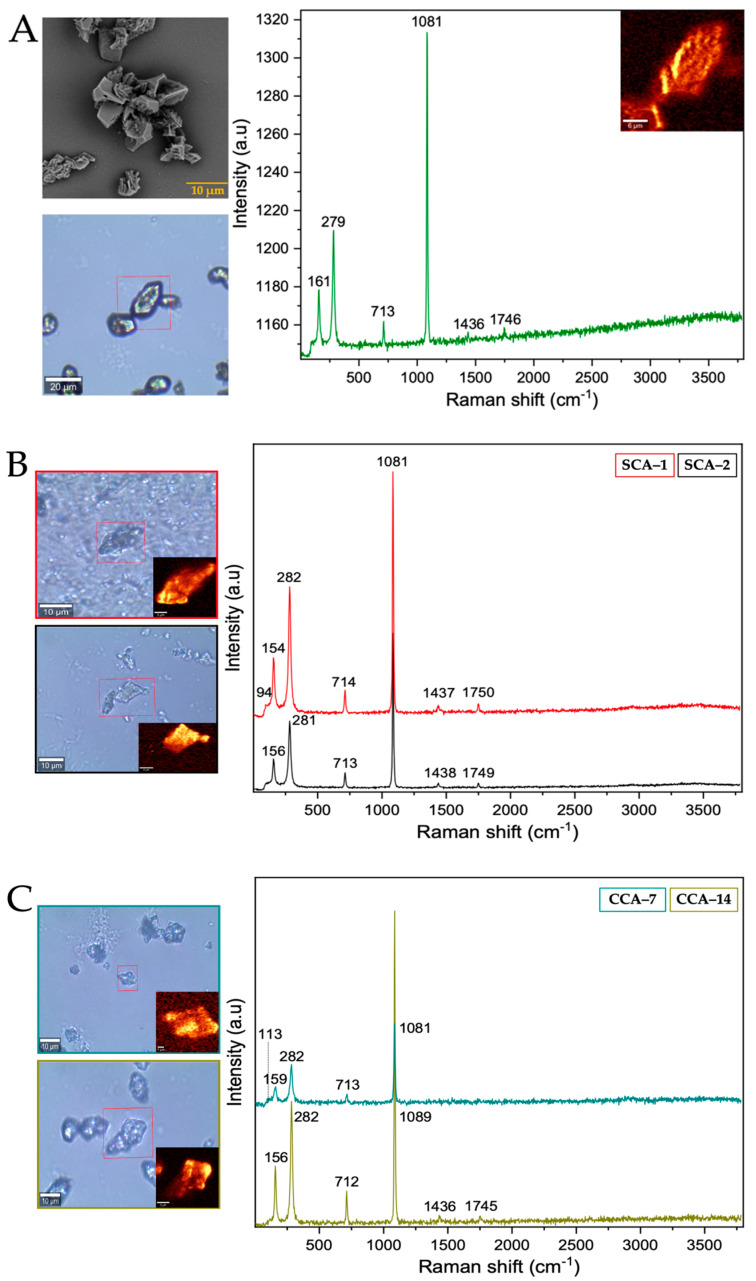
Raman spectra of calcium silica-carbonates synthesized. (**A**) Control; (**B**) Struthiocalcins; (**C**) Crococalcins from crocodile *acutus*. The blue images are optical images, and the smaller ones correspond to the mapping performed on the biomorphs.

From the Raman spectrum of the control (Table 1 and Figure 4A), the bands at 161, 279, 713, 1081, 1436, and 1748 cm^−1^ correspond to calcium carbonate in the form of trigonal calcite [35]. However, the bands obtained from the structures synthesized using intramineral proteins (Figure 4B,C and Figure S5A,B) did not present characteristic shifts and the synthesized calcium carbonate corresponds to the calcite polymorph. This result is probably an indication that the synthesis occurs spontaneously avoiding the interaction and influence of proteins on the growth and on the obtaining of other calcium carbonate polymorphs as has been observed in other investigations [36].

**Figure 5:**
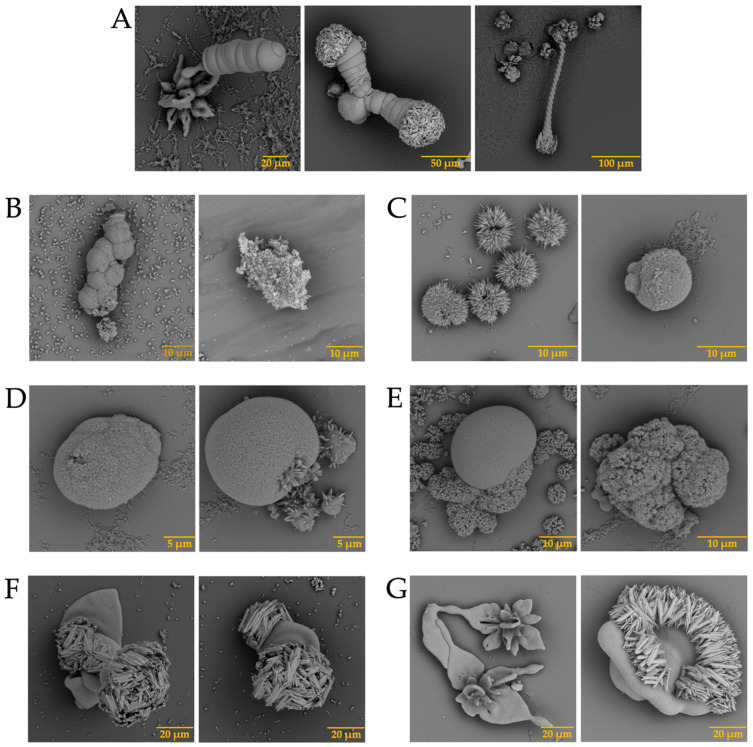

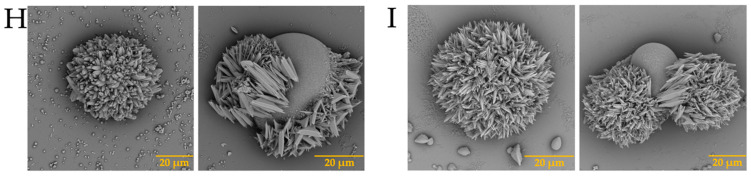
SEM micrographs of barium silica-carbonates structures in the presence of intramineral proteins. (**A**) Control; (**B**) SCA-1; (**C**) SCA-2; (**D**) DCA-1; (**E**) DCA-2; (**F**) CCA-7; (**G**) CCA-14; (**H**) CCM-1; (**I**) CCM-3.

In addition, barium biomorphs were also synthesized at room temperature in the presence and absence of intramineral proteins. According to Figure 5, the control presented flower, helix, and acicular morphologies. The structures obtained with the influence of intramineral proteins presented different morphologies compared to those observed in the control (Figure 5A). In the case of ratite birds (Figure 5B–E), the morphology observed was hemispherical with pointed needle-like ends, as well as other morphologies similar to those observed in spores and pollen [37]. When the structures obtained with the proteins isolated from crocodile eggshells were observed (Figure 5F–I). Spherical morphologies with needle-like columnar and acicular structures were obtained, where the morphology of these structures was very different from those observed in ratite birds. In the case of the CCA-14 protein, a flower-like structure resembling orchid petals was obtained (Figure 5G). All these structures presented a morphology very characteristic of those observed in some diatom microfossils [38], such as in the cells of the unicellular alga *Cladophora*, marine plankton, freshwater algae, and branched stems in the *Licmophora* mucilage [39].

**Figure 6:**
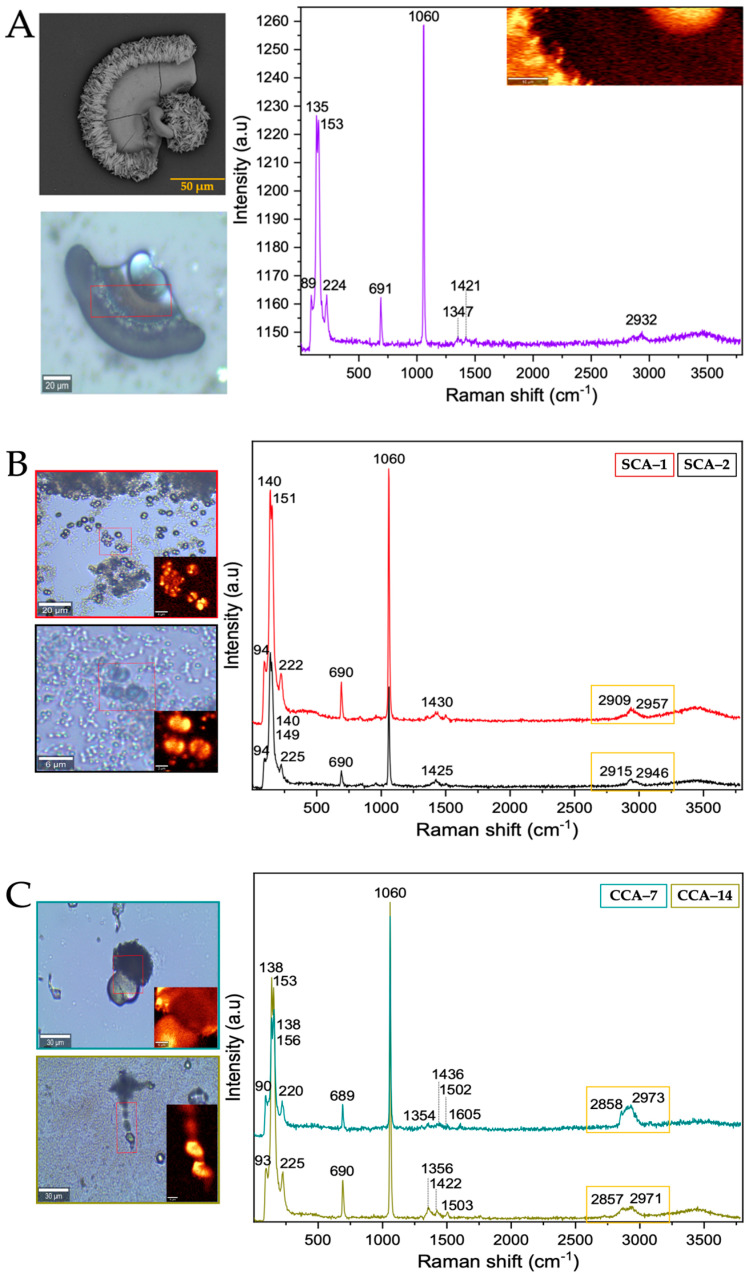
Raman spectra of barium silica-carbonates synthesized. (**A**) Control; (**B**) Struthiocalcins; (**C**) Crococalcins from crocodile *acutus*. The blue images are optical images, and the smaller ones correspond to the mapping performed on the biomorphs. The yellow box indicates the protein signal.

From the Raman spectra of the barium biomorphs, it was observed that the bands at 89, 135, 153, 224, 691, 1060, 1347, 1421, and 2932 cm^−1^ correspond to barium carbonate BaCO_3_ in the form of orthorhombic witherite (Table 2 and Figure 6A) [40]. In the samples synthesized in the presence of intramineral proteins, the spectra showed bands similar to those previously observed in the control (Figure 6B,C and Figure S6), but unlike the spectra of the calcium biomorphs, the spectra of the barium biomorphs showed bands between 2800 and 3000 cm^−1^, characteristic of biomolecule signals. This is an indication that the process is somewhat slower and allows for intramineral proteins to be involved in biomorph formation [35,36].

**Figure 7:**
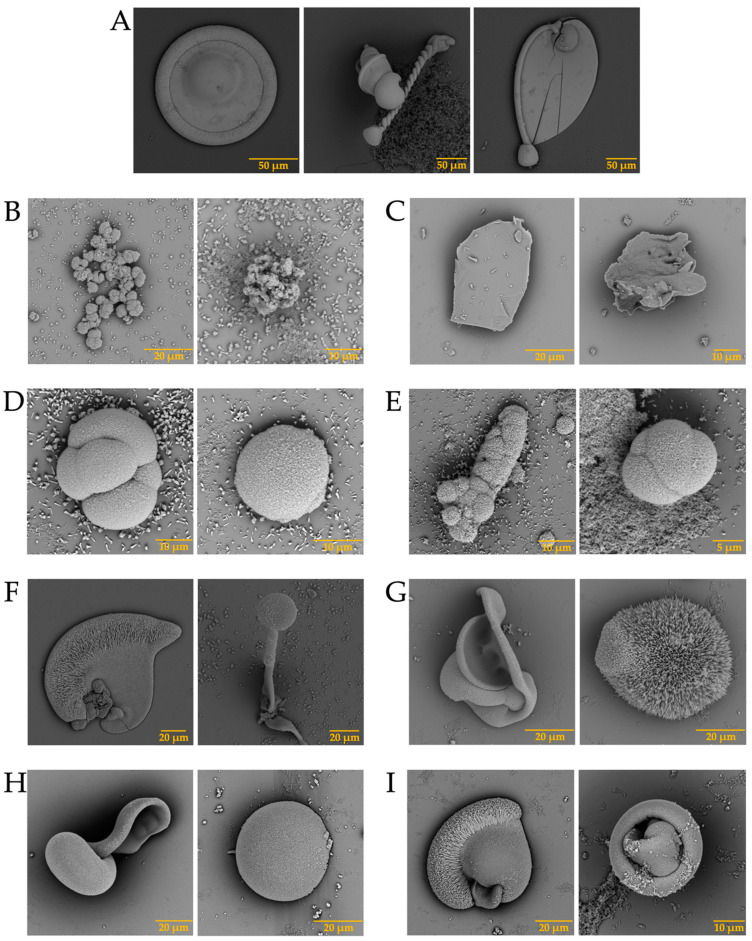
SEM micrographs of strontium silica-carbonates structures in the presence of intramineral proteins. (**A**) Control; (**B**) SCA-1; (**C**) SCA-2; (**D**) DCA-1; (**E**) DCA-2; (**F**) CCA-7; (**G**) CCA-14; (**H**) CCM-1; (**I**) CCM-3.

In the case of the strontium carbonate biomorphs, we obtained structures that had not been seen in previous cases. The structures observed in the control showed spherical morphologies such as those observed in diatoms from Little Round Lake in Ontario [41], and helical similar to plant leaves (Figure 7A). In the case of ostrich proteins (Figure 7B,C), the structures formed were similar to those observed in calcium biomorphs (Figure 3B,C). However, the influence of the proteins can be seen as the morphology of the biomorphs obtained is completely different from the control. In the tests where a greater influence of part of the intramineral proteins is seen, it corresponds to the emu and the two crocodile species. These morphologies have been observed in foraminifera, which correspond to eukaryotic organisms, and are considered the most important marine microfossils abundantly found in calcareous sediments [42]. Something similar is observed in the structures obtained with crocodile proteins. In the case of crocodile *acutus* (Figure 7F,G), the morphology shows a similarity with the morphologies present in spore microfossils [38]. In the case of crocodile *moreletti*, the structures had a similarity with the morphologies observed in diatoms, with a cleft in the center and spherical-like cells (Figure 7H,I) [38,39].

**Figure 8:**
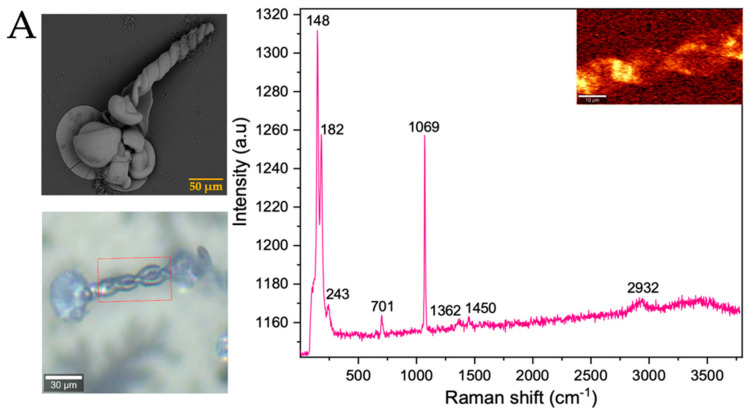

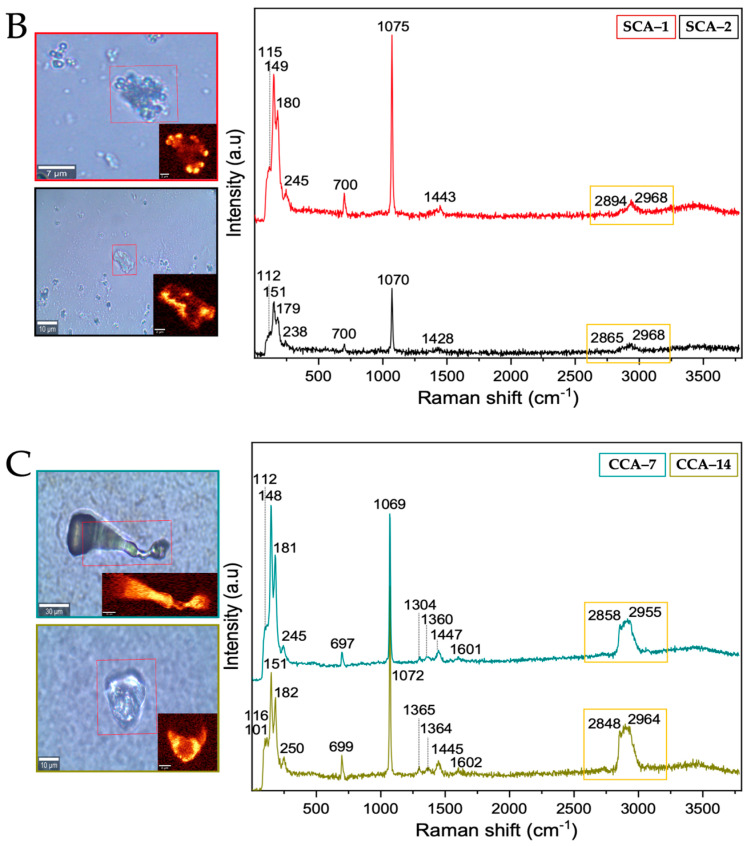
Raman spectra of strontium silica-carbonates synthesized. (**A**) Control; (**B**) Struthiocalcins; (**C**) Crococalcins from crocodile *acutus*. The blue images are optical images, and the smaller ones correspond to the mapping performed on the biomorphs. The yellow box indicates the protein signal.

After analyzing the morphology of these strontium biomorphs in the presence of proteins, we analyzed the Raman spectra, observing that the control presented bands at 148, 182, 243, 701, 1069, 1362, 1450, and 2932 cm^−1^, corresponding to orthorhombic SrCO_3_ stronthianite (Table 3 and Figure 8A) [12]. In the case of the biomorphs synthesized in the presence of proteins, they presented bands characteristic of strontium carbonate, similar to those observed in the control (Table 3, Figure 8 and Figure S7).

These results indicate that the synthesis of biomorphs in the presence of intramineral proteins is more ordered in the presence of barium and strontium (strontium predominates), allowing to obtain structures with unique and reproducible morphologies, similar to those observed in diatom and radiolarian microfossils. However, when the synthesis is carried out in the absence of proteins, the morphologies obtained in the structures are different from those obtained in the presence of biomolecules. These facts agree with the results previously published by other research groups, where they demonstrate the influence of peptides on the morphology of mineralized structures [43].

### 3.4. Elemental Analysis and Biomorphs Growth on Eggshell Membranes

**Figure 9:**
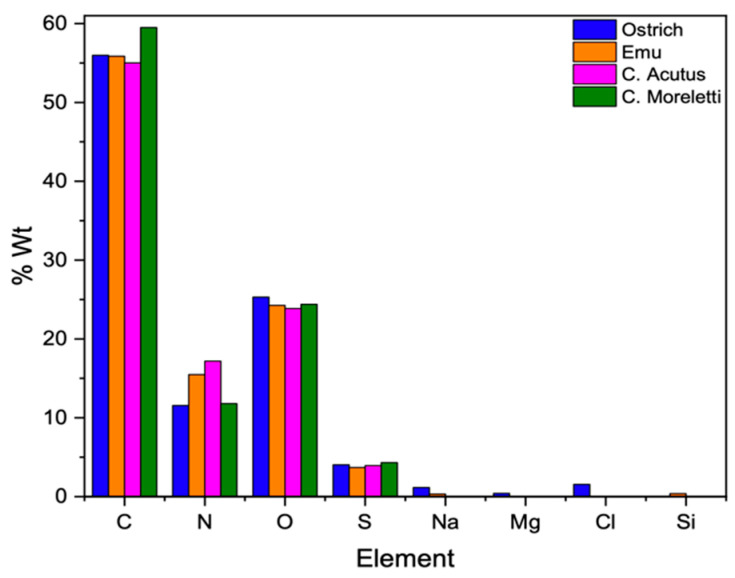
Elemental composition in molecular weight percentage of the membranes of the four species under study.

After the separation and cleaning of the membranes were completed, the EDS analysis was carried out in the area framed by the line of the membranes of the eggshells studied (Figure S8). In all the samples, elements such as carbon (C), nitrogen (N), oxygen (O), and sulfur (S) represented the highest percentage, being the main components of an organic system constituting a major part of life. In the case of ratite birds’ membranes, other elements such as sodium (Na) were observed in both samples. Magnesium (Mg) and chlorine (Cl) were observed only in the ostrich sample, whereas silicon (Si) was observed in the emu membrane (Figure 9 and Table S3). These trace elements, even if in low quantities, are essential in the functioning of a system that adapts or evolves according to the conditions of the habitat in which it develops [44,45,46].

**Figure 10:**
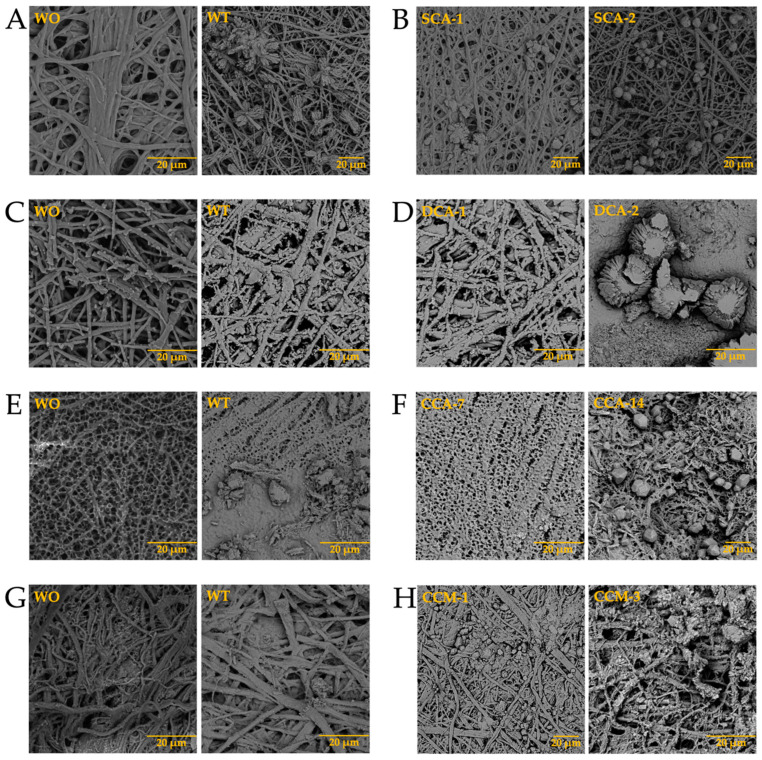
Growth of calcium silica-carbonate structures on eggshell membranes of ostrich (**A**,**B**), emu (**C**,**D**), crocodile *acutus* (**E**,**F**), and crocodile *moreletti* (**G**,**H**). WO: Without calcium carbonate; WT: With calcium carbonate; SCA: Struthiocalcin; DCA: Dromaiocalcin; CCA: Crocodile *acutus* crococalcin; CCM: Crocodile *moreletti* crococalcin.

Once the elements present in the membranes were identified, and considering that biomineralization is mediated by biomacromolecules, the synthesis of biomorphs was carried out in the absence and presence of intramineral proteins, using the membranes isolated from eggshells as scaffolds for structural growth. The first test performed was the growth of biomorphs on the untreated membranes, taking them as a control (Figure 10-WO). This WO acronym stands for without, while WT stands for with. Subsequently, the same procedure was carried out for the synthesis of calcium biomorphs, but in presence of intramineral proteins isolated from the eggshells of ratite birds and reptiles. From the first test performed with the ostrich membrane in the absence of protein (Figure 10A with WT), spherulitic structures were observed between the membranes and on the membrane fibers. In the presence of struthiocalcins (Figure 10B), SCA-1 presented a behavior similar to that observed in the absence of protein, while in the presence of SCA-2 the structures showed a spherulitic morphology. This type of morphology is very characteristic of structures involving biomacromolecules, as seen in studies developed in our research group [33]. It is worth mentioning that the structures obtained present a similarity with those found on the inner surface of the eggshell (Figure S9). These results are a starting point for understanding the process of eggshell formation in other species phylogenetically related to the chicken (further information is available in the literature). Continuing with the ratite birds, the emu membrane in the absence of protein did not present any structure of interest, but only precipitates around the membrane fibers (Figure 10C-WT). In the presence of dromaiocalcins, however, the results obtained were of great interest as it could be proved that DCA-1 plays the role of preparing the membrane while, at the same time, forms the nucleation sites for DCA-2 to later carry out the formation of the mammillary knobs, located in the inner part of the eggshell (Figure S9). The mammillary knobs are necessary in the formation of the mineral palisade which presents a columnar shape, giving way to the form of the pores and to the final structure of the eggshell [47]. Comparison of the results obtained in emu with those obtained in ostrich, easily leads us to think that the spherical structures formed in the presence of SCA-2 correspond to the mammillary knobs, needing a higher concentration of protein than the one used.

In the case of reptiles, the membrane of the crocodile *acutus* eggshell presented a morphology similar to that seen in rose spines (Figure 10E-WT), while the membrane of the crocodile *moreletti* eggshell are similar to those observed in the membranes of ratite birds (Figure 10G-WT). In the presence of CCA-14 proteins (Figure 10F) semi-spherical structures were observed, while in the presence of CCM-3 (Figure 10H) the structures presented a spherulitic morphology. However, in the presence of CCA-7 and CCM-1 proteins (Figure 10F,H), no structures with interesting morphologies were observed, but only precipitates covering the membrane fibers. This suggests that the lower molecular weight proteins probably play the role of initiating the biomineralization process while the higher molecular weight proteins play the role of carrying out the eggshell formation. Nevertheless, as we wanted to know the composition of the membranes and the structures obtained in the absence and presence of the intramineral proteins, we had to analyze them by FTIR, as in these cases it was difficult to do it by Raman due to the not neglectable fluorescence of the samples.

**Figure 11:**
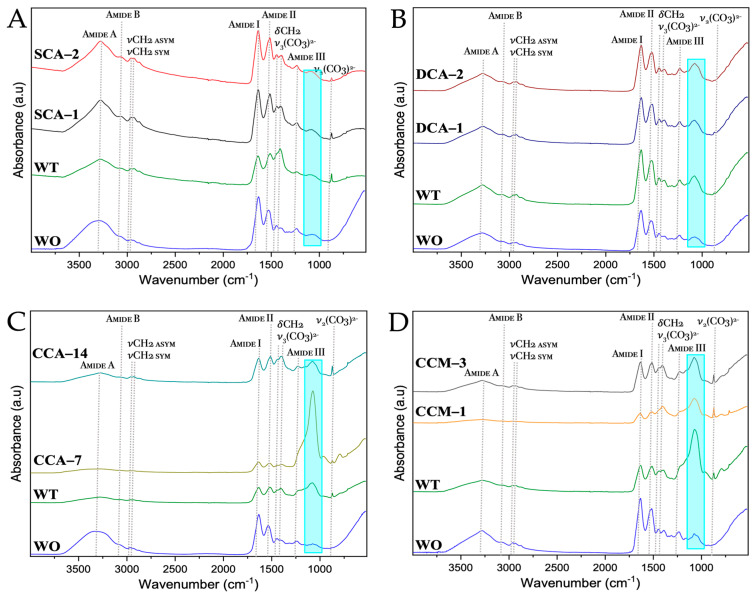
FTIR spectra of untreated membranes of ratite birds and reptiles before and after synthesis of calcium biomorphs. (**A**) Ostrich; (**B**) Emu; (**C**) Crocodile *acutus*; (**D**) Crocodile *moreletti*; WO: Without CaCO_3_; WT: With CaCO_3_; SCA: Struthiocalcin; DCA: Dromaiocalcin; CCA: Crocodile *acutus* crococalcin; CCM: Crocodile *moreletti* crococalcin. The blue box indicates an unidentified signal at the time of data collection.

From the FTIR spectra of the membranes of ratite birds and reptiles (Figure 11 and Tables S4–S7), we observed practically the same signals where only just the absorption of each of them varied. The signals at wavenumbers between 2500 and 3500 cm^−1^ corresponded both to proteins of amide group A and group B. Amide group A is characterized by hydrogen bond vibrations with lengths from 2.69 to 285 Å, whereas amide group b is characterized by the stretching vibrations of the amine group (-NH). The symmetric (*sym*) and asymmetric (*asym*) strain signals (ν) of the methyl group (-CH_2_) were also observed. At lower wavenumbers, in the amide group I (~1730–1580 cm^−1^) characterized by high absorption focused on the vibrations of the carbonyl (-C=O) and methyl amine (-C-N), we found groups of the main chain of amino acids [48,49].

The other group corresponding to amino acids is amide II (~1580–1450 cm^−1^), focused on the bending vibrations (δ) of the amine (-NH) and stretching of the methyl amine (-C-N) and dimethyl (-C-C) groups. Next, we see the bending vibration signal of the methyl group (-CH_2_) at ~1480–1430 cm^−1^ and that of the ν_3_ mode corresponding to the carbonate ((CO_3_)^2−^) between ~1420–1350 cm^−1^. Subsequently, we see the amide group III which gives information about the vibrations of the hydrogen bonds of the amino acids and is found at wavenumbers from ~1450–1200 cm^−1^ [50,51]. Finally, the ν_2_ mode of carbonate ((CO_3_)^2−^) is found at a wavenumber about ~870 cm^−1^, which is not observed in the untreated membranes, giving us a sign that the calcium carbonate mineral of the mineral phase of the eggshell has indeed been completely dissolved during the cleaning with acetic acid and 3-mercaptopropionic acid (see Section 2). In addition, a signal was identified at ~1080 cm^−1^ (blue box) corresponding to the asymmetric stretching vibrational mode of silicon oxide (Si-O-Si) [52]. This was confirmed by the elemental composition of the membranes, where silicon could be identified in the emu sample (Figure 9).

### 3.5. Biocalcification and Biosilicification of Membranes and Calcium Biomorphs Production

**Figure 12:**
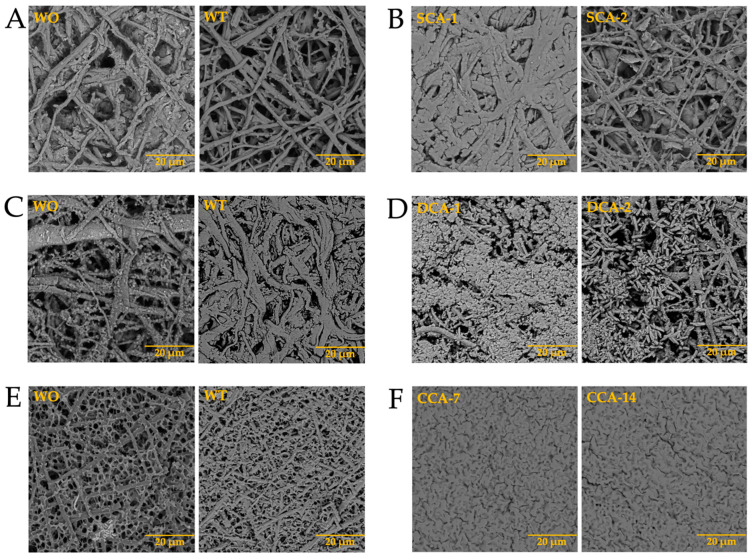

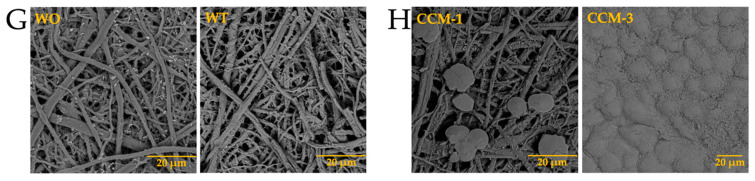
Growth of barium silica-carbonate structures on eggshell biocalcified membranes of ostrich (**A**,**B**), emu (**C**,**D**), crocodile *acutus* (**E**,**F**), and crocodile *moreletti* (**G**,**H**). WO: Without calcium carbonate; WT: With calcium carbonate; SCA: Struthiocalcin; DCA: Dromaiocalcin; CCA: Crocodile *acutus* crococalcin; CCM: Crocodile *moreletti* crococalcin.

After analyzing the behavior of intramineral proteins in the growth of calcium biomorphs, we performed the synthesis of calcium carbonate on membranes treated with calcium phosphate (biocalcification). In Figure 12 we observed that during the growth of calcium carbonate biomorphs (WO and WT) there was no characteristic difference in the membranes treated with phosphate. However, during synthesis in the presence of intramineral proteins, there was a small influence of phosphate present in the treated membranes of both ratite birds and reptiles. Among the structures observed in the treated ostrich membrane, a growth of small structures in the presence of SCA-2 (Figure 12B) was present very similar to those observed in the inner part of the eggshell (Figure S9A) and in the case of SCA-1 (Figure 12B), the formation of precipitates allowed us to observe a morphology similar to those found in the outer part of the shell (Figure S9A).

Similar results were observed in the structures obtained during the synthesis of calcium carbonate in the presence of dromaiocalcins (Figure 12D), where the structures are slightly smaller compared to those observed in the presence of struthiocalcins. They present similarities with the outer part of the eggshell in the case of DCA-1 and with the inner part in the case of DCA-2 (Figure S9B). The opposite case was observed in the presence of crococalcins (Figure 12F,H). In the presence of CCA (Figure 12F), the formation of precipitates was observed, giving the membrane a smooth appearance very similar to that observed on the outer part of the crocodile *acutus* eggshell (Figure S9C). While in the case of the CCM (Figure 12H), hemispherical structures were formed, but in the presence of CCM-1, these structures were obtained scattered over the entire surface of the membrane, giving an appearance similar to that observed on the outer part and, at the same time, that of the inner part, since there was no total coverage of the membrane as observed in the case of CCM-3 which imitates the morphology of the outer part of the eggshell (Figure S9D).

**Figure 13:**
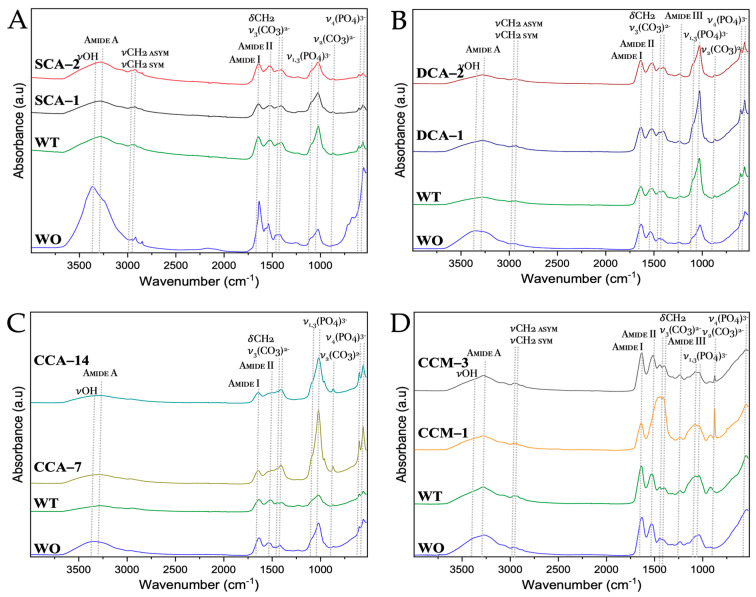
FTIR spectra of biocalcified membranes of ratite birds and reptiles before and after synthesis of calcium biomorphs. (**A**) Ostrich; (**B**) Emu; (**C**) Crocodile *acutus*; (**D**) Crocodile *moreletti*; WO: Without CaCO_3_; WT: With CaCO_3_; SCA: Struthiocalcin; DCA: Dromaiocalcin; CCA: Crocodile *acutus* crococalcin; CCM: Crocodile *moreletti* crococalcin. The blue box indicates an unidentified signal at the time of data collection.

As in the case of the untreated membranes, the biocalcified membranes were analyzed by FTIR and the signals corresponding to the hydroxyapatite calcium phosphate present in the membrane fibers were observed (Figure 13 and Tables S8–S11). One of these signals corresponds to the tension (ν) of the hydroxyl (-OH), which vibrates at ~3300 cm^−1^, it is typical of biogenic hydroxyapatite found in membrane [50], where the collagen presence lowers the (-OH) infrared peak of mineral hydroxyapatite of 3570 cm^−1^. The other signals correspond to the vibration modes [ν1+ν3] and [ν2+ν4] of the phosphate group ((PO_4_)^3−^), which were observed at ~950–1100 cm^−1^ and ~520–650 cm^−1^, respectively [53,54,55].

**Figure 14:**
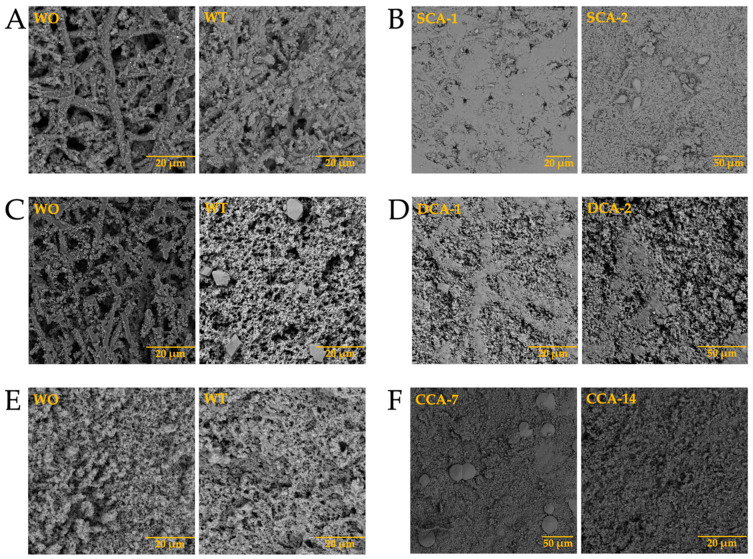

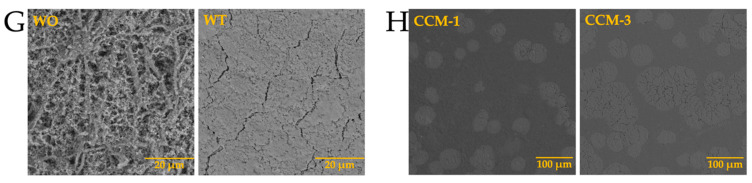
Growth of barium silica-carbonate structures on eggshell biosilicified membranes of ostrich (**A**,**B**), emu (**C**,**D**), crocodile *acutus* (**E**,**F**), and crocodile *moreletti* (**G**,**H**). WO: Without calcium carbonate; WT: With calcium carbonate; SCA: Struthiocalcin; DCA: Dromaiocalcin; CCA: Crocodile *acutus* crococalcin; CCM: Crocodile *moreletti* crococalcin.

After observing the structures obtained in the biocalcified membranes, we continued with the growth of calcium carbonate biomorphs using biosilicified membranes. In this case, we observed the formation of precipitates immersed in the fibers of the membranes (Figure 14-WO). When performing the growth of biomorphs in the absence of intramineral proteins, we observed the formation of small structures characteristic of calcium carbonate crystals in the membranes of ratite birds (Figure 14A,C-WT), while in the membranes of reptiles (Figure 14E,G-WT), we obtained precipitates mainly between the fibers.

From the tests carried out in the presence of intramineral proteins, no structures with defined morphologies were formed in the membranes of ratite birds (Figure 14B,D), although we were able to identify that the form in which the precipitate was deposited, has similarities to the intermediate phase of the eggshell. This can be observed in the lateral image of Figure S9A,B. This intermediate phase corresponds to the palisade layer responsible for the formation of the pores and, in turn, the shape of the eggshell. In the membrane of crocodile *acutus* eggshell (Figure 14F) a behavior similar to that observed in ratite birds was observed. However, we were unable to determine whether it corresponded to the lateral phase, since this eggshell presents a laminar structure assembled on top of each other (Figure S9C). In the crocodile *moreletti* eggshell (Figure 14H), we observed the formation of a kind of circular islands, similar to those observed in the lateral phase of the eggshell (Figure S9D), leading us to believe that in the case of crocodile *acutus*, a similar behavior would occur, where the precipitates obtained would be the same as those contained in the lamellae located in the central part of the eggshell.

**Figure 15:**
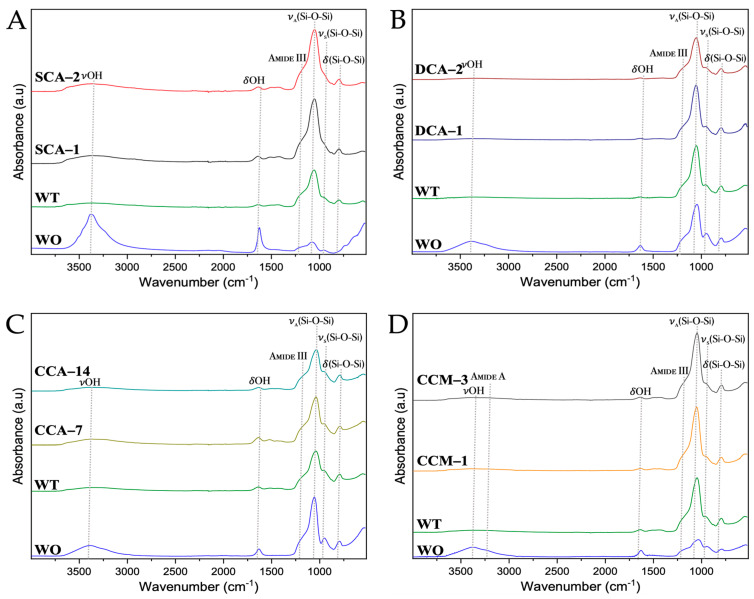
FTIR spectra of biosilicified membranes of ratite birds and reptiles before and after synthesis of calcium biomorphs. (**A**) Ostrich; (**B**) Emu; (**C**) Crocodile *acutus*; (**D**) Crocodile *moreletti*; WO: Without CaCO_3_; WT: With CaCO_3_; SCA: Struthicalcin; DCA: Dromaiocalcin; CCA: Crocodile *acutus* crococalcin; CCM: Crocodile *moreletti* crococalcin. The blue box indicates an unidentified signal at the time of data collection.

From the FTIR spectra of the biosilicified membranes, most of the signals were identified in the previous spectra, whereas among signals corresponding to proteins, only the signal corresponding to amide III was observed (Figure 15 and Tables S12–S15). This result is a possible indication that the silica present in the membrane fibers acts as a barrier, protecting proteins, which are the main component of the membrane and of the biomorphs. In addition, a hydroxyl (-OH) bending signal (δ) was observed at wavenumber ~1636 cm^−1^ corresponding to the water molecules present in the membranes. The characteristic signals of silicon oxide (Si-O-Si) were observed at wavenumbers of ~1100 cm^−1^ and ~950 cm^−1^ for asymmetric (*asym*) and symmetric (*sym*) tension (ν) and at ~830 cm^−1^ for bending signals (δ) [52].

## 4. Conclusions

Nine proteins were isolated from the eggshells of ratite birds and reptiles, eight of which were used for the growth of calcium, barium, and strontium carbonate biomorphs, using glass and membranes extracted from the eggshells as scaffolds. From the growth of the biomorphs developed on glass, a strong influence of the proteins on the microstructure and stability of the carbonates was identified. In the presence of the membrane, the observed structures presented a similarity to the structures found naturally in the eggshell of each species under study, which have unique and defined morphologies that are almost impossible to reproduce without intramineral proteins. These results contribute to the understanding of how and which proteins are responsible for the formation of each of the eggshell phases. However, chemical mechanisms to fully elucidate the function of these proteins in calcium carbonate biomineralization are still missing. Currently, we are performing experiments to understand the role of proteins and genes involved in all those processes.

## Figures and Tables

**Table 1:** Raman shift observed in calcium silica-carbonates in the presence of intramineral proteins from ratite birds and reptiles *.

Control	SCA-1	SCA-2	DCA-1	DCA-2	CCA-7	CCA-14	CCM-1	CCM-3
--	94	--	108	115	113	--	108	--
161	154	156	158	153	159	156	154	153
279	282	281	283	279	282	282	280	279
713	714	713	713	713	713	712	711	711
1081	1081	1081	1089	1090	1081	1089	1081	1081
1436	1437	1437	1443	--	--	1436	1430	1436
1748	1750	1749	1752	--	--	1745	1755	1745

* The shift is given in cm^−1^.

**Table 2:** Raman shift observed in barium silica-carbonates in the presence of intramineral proteins from ratite birds and reptiles *.

Control	SCA-1	SCA-2	DCA-1	DCA-2	CCA-7	CCA-14	CCM-1	CCM-3
89	94	94	88	93	90	93	91	91
135	140	140	134	135	138	138	136	136
153	151	149	150	150	153	156	155	155
224	222	225	214	224	220	225	224	222
691	690	690	689	688	689	690	691	690
1060	1060	1060	1057	1056	1060	1060	1060	1060
1347	--	--	--	--	1354	1356	1354	1351
1421	1430	1425	1428	1436	1436	1422	1424	1433
--	--	--	--	--	1502	1503	--	--
--	--	--	--	--	1605	--	--	--
--	2909	2915	2848	2841	2858	2857	2852	2850
2932	2957	2946	2947	2947	2973	2971	2944	2942

* The shift is given in cm^−1^.

**Table 3:** Raman shift observed in strontium silica-carbonates in the presence of intramineral proteins from ratite birds and reptiles *.

Control	SCA-1	SCA-2	DCA-1	DCA-2	CCA-7	CCA-14	CCM-1	CCM-3
--	115	112	111	114	112	116	119	115
148	149	151	145	144	148	151	151	148
182	180	179	177	177	181	182	182	180
243	245	238	234	246	245	250	243	246
701	700	700	697	696	679	699	699	698
1069	1075	1070	1067	1067	1069	1072	1070	1075
--	--	--	--	--	1304	1305	1297	--
1362	--	--	1354	--	1360	1364	--	--
1450	1443	1428	1443	--	1447	1445	1440	1446
--	--	--	--	1605	1601	1602	1458	--
--	2894	2865	2847	--	2858	2848	2853	2848
2932	2968	2968	2943	2932	2955	2964	2945	2967

* The shift is given in cm^−1^.

## Data Availability

Data are contained within the article and Supplementary Materials.

## Supplementary Materials

The following supporting information can be downloaded at: https://www.mdpi.com/article/10.3390/membranes13110869/s1, Table S1: Fractions collected from injections made with 50 mM sodium citrate + 150 mM NaOH pH 4.0 at a flow rate of 0.5 mL/min. Fractions were collected every 1 mL and the volume in mL of the collected fractions is given. Fxn: Fraction; Figure S1: The fractions taken for the SDS-PAGE gel from ostrich (A) and emu (B) are listed in Table S1 with their respective volumes. The molecular weight marker (MM) corresponds to Prestained Protein Ladder (10–245 kDa); Table S2: Fractions collected from injections made with 50 mM sodium citrate + 150 mM NaOH pH 4.0 at a flow rate of 0.5 mL/min. Fractions were collected every 1 mL and the volume in mL of the collected fractions is given. Fxn: Fraction; Figure S2: The fractions taken for the SDS-PAGE gel from crocodile *acutus* (A) and crocodile *moreletti* (B) are listed in Table S2 with their respective volumes. The molecular weight marker (MM) corresponds to Prestained Protein Ladder (10–245 kDa); Figure S3: Mass spectra of intramineral proteins SCA-1 (A), SCA-2 (B), DCA-1 (C), and DCA-2 (D); Figure S4: Mass spectra of intramineral proteins CCA-7 (A), CCA-14 (B), CCM-1 (C), CCM-2 (D), and CCM-3 (E); Figure S5: Raman spectra of calcium silica-carbonates synthesized. A: Dromaiocalcins; B: Crococalcins from crocodile *moreletti*. The blue images are optical images, and the smaller ones correspond to the mapping performed on the biomorphs; Figure S6: Raman spectra of barium silica-carbonates synthesized. A: Dromaiocalcins; B: Crococalcins from crocodile *moreletti*. The blue images are optical images, and the smaller ones correspond to the mapping performed on the biomorphs. The yellow box indicates the protein signal; Figure S7: Raman spectra of strontium silica-carbonates synthesized. A: Dromaiocalcins; B: Crococalcins from crocodile *moreletti*. The blue images are optical images, and the smaller ones correspond to the mapping performed on the biomorphs. The yellow box indicates the protein signal; Table S3: Elemental percentage present in the membranes of the ratite birds and crocodile’s eggshell; Figure S8: SEM-EDS images of the area outlined in white corresponding to the analysis performed on the eggshells’ membranes of ratite birds and crocodiles. A: Ostrich; B: Emu; C: C. *acutus*; D: C. *moreletti*; Figure S9: SEM images of ostrich (A), emu (B), crocodile *acutus* (C), and crocodile *moreletti* (D) eggshells. Ext: Outer part; Int: Inner part; Lat: Lateral/Intermediate section; Table S4: Assignment of the FTIR vibrations of untreated membranes of ostrich before and after synthesis of calcium biomorphs. WO: Without CaCO_3_; WT: With CaCO_3_; SCA: Struthicalcin; Table S5: Assignment of the FTIR vibrations of untreated membranes of emu before and after synthesis of calcium biomorphs. WO: Without CaCO_3_; WT: With CaCO_3_; DCA: Dromaiocalcin; Table S6: Assignment of the FTIR vibrations of untreated membranes of crocodile *acutus* before and after synthesis of calcium biomorphs. WO: Without CaCO_3_; WT: With CaCO_3_; CCA: Crococalcin; Table S7: Assignment of the FTIR vibrations of untreated membranes of crocodile *moreletti* before and after synthesis of calcium biomorphs. WO: Without CaCO_3_; WT: With CaCO_3_; CCM: Crococalcin; Table S8: Assignment of the FTIR vibrations of biocalcified membranes of ostrich before and after synthesis of calcium biomorphs. WO: Without CaCO_3_; WT: With CaCO_3_; SCA: Struthicalcin; Table S9: Assignment of the FTIR vibrations of biocalcified membranes of emu before and after synthesis of calcium biomorphs. WO: Without CaCO_3_; WT: With CaCO_3_; DCA: Dromaiocalcin; Table S10: Assignment of the FTIR vibrations of biocalcified membranes of crocodile *acutus* before and after synthesis of calcium biomorphs. WO: Without CaCO_3_; WT: With CaCO_3_; CCA: Crococalcin; Table S11: Assignment of the FTIR vibrations of biocalcified membranes of crocodile *moreletti* before and after synthesis of calcium biomorphs. WO: Without CaCO_3_; WT: With CaCO_3_; CCM: Crococalcin; Table S12: Assignment of the FTIR vibrations of biosilicified membranes of ostrich before and after synthesis of calcium biomorphs. WO: Without CaCO_3_; WT: With CaCO_3_; SCA: Struthiocalcin; Table S13: Assignment of the FTIR vibrations of biosilicified membranes of emu before and after synthesis of calcium biomorphs. WO: Without CaCO_3_; WT: With CaCO_3_; SCA: Dromaiocalcin; Table S14: Assignment of the FTIR vibrations of biosilicified membranes of crocodile *acutus* before and after synthesis of calcium biomorphs. WO: Without CaCO_3_; WT: With CaCO_3_; CCA: Crococalcin; Table S15: Assignment of the FTIR vibrations of biosilicified membranes of crocodile *moreletti* before and after synthesis of calcium biomorphs. WO: Without CaCO_3_; WT: With CaCO_3_; CCA: Crococalcin.

## References

[1] Weiner S., Dove P.M. (2003). An overview of biomineralization processes and the problem of the vital effect. Rev. Mineral. Geochem..

[2] Weiner S. (2008). Biomineralization: A structural perspective. J. Struct. Biol..

[3] Reznikov N., Steele J.A.M., Fratzl P., Stevens M.M. (2016). A materials science vision of extracellular matrix mineralization. Nat. Rev. Mater..

[4] Mann S. (1983). Mineralization in Biological Systems. Inorganic Elements in Biochemistry: Structure and Bonding.

[5] Gower L. (2008). Biomimetic model systems for investigating the amorphous precursor pathway and its role in biomineralization. Chem. Rev..

[6] Guatron J., Stapane L., Le Roy N. (2021). Avian eggshell biomineralization: An update on its structure, mineralogy, and protein tool kit. BMC Mol. Cell. Biol..

[7] Suzuki M., Nahasawa H. (2013). Mollusk shell structures and their formation mechanism. Can. J. Zool..

[8] Mikhailov K.E. (1999). Fossil and Recent Eggshell in Amniotic Vertebrates: Fine Structure, Comparative.

[9] Yang W., Lopez P.J., Rosengarten G. (2011). Diatoms: Self-assembled silica nanostructures, and templates for bio/chemical sensors and biomimetic membranes. Analyst.

[10] Zheng W., Zhang W., Jiang X. (2010). Biomimetic collagen nanofibrous materials for bone tissue engineering. Adv. Eng. Mater..

[11] Dauphin Y., Luquet G., Perez-Huerta A., Salomé M. (2018). Biomineralization in modern avian calcified eggshells: Similarity versus diversity. Connect Tissue Res..

[12] Dauphin Y., Cuif J.P., Salomé M., Susini J., Williams C.T. (2006). Microstructure, and chemical composition of giant avian eggshells. Anal. Bioanalytical. Chem..

[13] Hincke M.T., Nys Y., Gautron J., Mann K., Rodriguez-Navarro A.B., McKee M.D. (2012). The eggshell: Structure, composition, and mineralization. Front. Biosci..

[14] Gautron J., Murayama E., Vignal A., Morisson M., McKee M., Rehault S., Hincke T.M. (2007). Cloning of ovocalyxin-36, a novel chicken eggshell protein related to lipopolysaccharide-binding proteins, bactericidal permeability-increasing proteins, and plink family proteins. J. Biol. Chem..

[15] Freeman C.L., Harding J.H., Quigely D., Rodger P.M. (2011). Simulations of Ovocleidin-17 binding to calcite surfaces and its implications for eggshells formation. J. Phys. Chem. C.

[16] Reyes-Grajeda J.P., Juaregui-Zuniga D., Rodriguez-Romero A., Hernandez-Santoyo A., Bolano-Garcia V.M., Moreno A. (2002). Crystallization, and preliminary X-ray analysis of ovocleidin-17 a major protein of the Gallus gallus eggshell calcified layer. Prot. Pep. Lett..

[17] Mann K., Siedler F. (2008). The amino sequence of ovocledin-17, a major protein fo the avian eggshell calcified layer. IUBMB Life.

[18] Lakshminarayanan R., Kini R.M., Valiyaveettil S. (2002). Investigation of the role of ansocalcin in the biomineralization in goose eggshell matrix. Proc. Natl. Acad. Sci. USA.

[19] Mann K., Siedler F. (2006). Amino acids sequence and phosphorylation sites of emu and rhea eggshell c-type lectin-like proteins. Comp. Biochem. Physiol. B Biochem. Mol. Biol..

[20] Reyes-Grajeda J.P., Marín-Garcaí L., Stojanoff V., Moreno A. (2007). Purification, crystallization, and preliminary X-ray analysis of struthiocalcin-1 from ostrich (*Struthio camelus*) eggshell. Acta Cryst..

[21] Eiblmeier J., Dankesreiter S., Ptitzner A., Schmalz G., Kellermeier M. (2014). Crystallization of mixed alkaline-earth carbonates in silica solution at high pH. Cryst. Growth Des..

[22] Kellermeier M., Melero-García E., Glaab F., Eblmeier J., Kienle L., Rachel R., Kunz W., García-Ruiz J.M. (2012). Growth behavior and kinetics of self-assembled silica-carbonate biomorphs. Chem. Eur. J..

[23] Virgen-Ortiz J.J., Ibarra-Junquera V., Osuna-Casto J.A., Escalante-Minakata P., Mancilla-Margalli N.A., Ornelas-Paz J.J. (2012). Methos to concentrate protein solutions based on dialysis-freezing centrifugation: Enzyme applications. Anal. Biochem..

[24] Noorduin W.L., Grinthal A., Mahadevan L., Aizenberg J. (2013). Rationally designed complex, hierarchical microarchitectures. Science.

[25] Li N., Niu L.N., Qi Y.P., Yiu C.K.Y., Ryou H., Arola D.D., Chen J.H., Pashley D.H., Tay F.R. (2011). Subtleties of biomineralization by manipulation of the eggshell membrane. Biomaterials.

[26] Ruiz-Arellano R.R., Moreno A. (2014). Obtainment of spherical-shaped calcite crystals induced of intramineral proteins isolated from eggshells of ostrich and emu. Cryst. Growth Des..

[27] Mann K. (2004). Identification of the major proteins of the organic matrix of emu (*Dromaius novaehollandiae*) and rhea (*Rhea americana*) eggshell calcified layer. Br. Poult. Sci..

[28] Cho Y.T., Su H., Huang T.L., Chen H.C., Wu W.K., Wu P.C., Wu D.C., Shiea J. (2013). Matrix-assisted lase desorption ionization/time-of-flight mass spectrometry for clinical diagnosis. Clin. Chim. Acta.

[29] Schiller J., Arnhold J., Benard S., Müller M., Reichi S., Arnold K. (1999). Lipid analysis by matrix-assisted lase desorption and ionization mass spectrometry: A methodological approach. Anal. Biochem..

[30] Legorreta-Flores A., Davila A., Velásquez-Gonzáles O., Ortega E., Ponce A., Castillo-Michel H., Reyes-Grajeda J.P., Hernández-Rivera R., Cuéllar-Cruz M., Moreno A. (2018). Calcium carbonate crystals shapes mediated by intramineral proteins from eggshells of ratite birds and crocodiles. Implications to the eggshell’s formation of a dinosaur of 70 million years old. Cryst. Growth Des..

[31] Schopf J.W. (1993). Microfossils of the early archean apex chert: New evidence of the antiquity of life. Science.

[32] Panheleux M., Nys Y., Williams J., Gautron J., Boldicke T., Hincke M.T. (2000). Extraction and quantification by ELISA of eggshell organic matrix proteins (ovocleidin-17, ovalbumin, ovotranferrin) in shell from young and old hens. Poult. Sci..

[33] Rodríguez-Navarro A., Kalin O., Nys Y., García-Ruiz J.M. (2002). Influence of the microstructure on the shell strength of eggs laid by hens of different ages. Br. Poult. Sci..

[34] Carteret C., Dandeu A., Moussaoui S., Muhr H., Humbert B., Plasari E. (2009). Polymorphism studied by lattice phonon Raman spectroscopy and statistical mixture analysis method. Application to calcium carbonate polymorphs during batch crystallization. Cryst. Growth Des..

[35] Cuéllar-Cruz M., Moreno A. (2019). The role of calcium and strontium as the most dominant elements during combinations of different alkaline earth metals in the synthesis of crystalline silica-carbonate biomorphs. Crystals.

[36] Opel J., Wimmer F.P., Kellermeier M., Cölfen H. (2016). Functionalisation of silica-carbonate biomorphs. Nanoscale Horiz..

[37] Rolleri C.H., Lavalle M.C., Mengascini A., Rodríguez M. (2003). Sistemática de los helechos maratiáceos (Marattiales-Marattiaceae). Revista del Museo de la Plata, Universidad Nacional de la Plata, Facultad de Ciencias Naturales y Museo. Botánica.

[38] Sims P.A., Mann D.G., Medlin L.K. (2006). Evolution of the diatoms: Insights from fossil, biological and molecular data. Phycologia.

[39] Round F.E., Crawford R.M., Mann D.G. (1990). The Diatoms. Biology & Morphology of the Genera.

[40] Rouillard J., García-Ruiz J.M., Gong J., Van Zuilen M.A. (2018). A morphogram for silica-whiterite biomorphs and its application to mircofossil identication in the early earth rock record. Giobiology.

[41] Dixit S.S., Smol J.P., Kingston J.C. (1992). Diatoms: Powerful indicators of environmental change. Environ. Sci. Technol..

[42] Quinn P. (2008). The occurrence and research potential of microfossils in inorganic archeological materials. Geoarchaeology.

[43] DeOliveira D.B., Laursen R.A. (1997). Control of calcite crystal morphology by a peptide designed to bind to a specific surface. J. Am. Chem. Soc..

[44] Williams R.J.P., Fraústo da Silva J.J.R. (1999). Bringing Chemistry to Life: From Matter to Man.

[45] Fraústo da Silva J.J.R., Williams R.J.P. (1991). The Biological Chemistry of the Elements: The Inorganic Chemistry of Life.

[46] Turner D.R., Whitfield M., Dickson A.G. (1981). The Equilibrium Speciation of Dissolved Components in Fresh-Water and Seawater at 25 °C and 1 Atm Pressure. Geochim. Cosmochim. Acta.

[47] Arias J.L., Fink D.J., Xiao S.Q., Heuer A.H., Caplan A.I. (1993). Biomineralization and eggshells: Cell-mediated acellular compartments of mineralized extracellular matrix. Int. Rev. Cytol..

[48] Byler D.M., Susi H. (1986). Examination of the secondary structure of proteins by deconvolved FTIR spectra. Biopolymers.

[49] Kong J., Yu S. (2007). Fourier transform infrared spectroscopic analysis of protein secondary structures. Acta Biochim. Biophys. Sin..

[50] Elejalde-Cadena N.R., Estevez-Espinoza J.O., Torres-Costa V., Ynsa M.D., García-López G., Moreno A. (2021). Molecular analysis and examination of posible intramineral proteins of dinosaur eggshells collected in El Rosario, Baja California. ACS Earth Space Chem..

[51] De Meutter J., Goormaghtigh E. (2021). FTIR imaging of protein microarrays for high throughput secondary structure determination. Anal. Chem..

[52] El-Fattah M.A., El-Saeed A.M., El-Ghazawy R.A. (2019). Chemical interaction of different sized fumed silica with epoxy via ultrasonication for improved coating. Prog. Org. Coat..

[53] Mendes L.C., Ribeiro G.L., Marques R.C. (2012). In situ hydroxyapatite synthesis: Influence of collagen on its structural and morphological characteristic. Mater. Sci. Appl..

[54] El Khouri A., Zegzouti A., Elaatmani M., Capitelli F. (2019). Bismuth-substituted hdroxyapatite ceramics synthesis: Morphological, structural, vibrational, and dielectric properties. Inorg. Chem. Commun..

[55] Cancelliere R., Rea G., Micheli L., Mantegazza P., Bauer E.M., El Khouri A., Tempesta E., Altomare A., Capelli D., Capitelli F. (2023). Electrochemical and structural characterization of lanthanum-doped hydroxyapatite: A promising material for sensing applications. Materials.

