# Supplementary material for: Influence of Intramineral Proteins on the Growth of Carbonate Crystals Using as a Scaffold Membranes of Ratite Birds and Crocodiles Eggshells
Source: Membranes (Basel). 2023 Nov 1;13(11):869. doi: 10.3390/membranes13110869 (PMC10672969; doi:10.3390/membranes13110869)
Supplement: Supplementary file 1 [file membranes-13-00869-s001.zip › membranes-2693847-supplementary.pdf]

membranes

Supporting Information

Influence of Intramineral Proteins on the Growth of Carbonate Crystals Using as a Scaffold Membranes of Ratite Birds and Crocodiles Eggshells

Nerith R. Elejalde-Cadena [1,2], Denisse Hernández [2], Francesco Capitelli [3], Selene R. Islas [4], Maria J. Rosales-Hoz [5], Michele Zema [6,7], Serena C. Tarantino [8,9], Dritan Siliqi [7,*] and Abel Moreno [2,*]

[1] Institute of Physics, National Autonomous University of Mexico, Circuito de la Investigación Científica s/n, Ciudad Universitaria, Ciudad de Mexico 045010, Mexico; rocioec@fisica.unam.mx
[2] Institute of Chemistry, National Autonomous University of Mexico, Av. Universidad 3000, Ciudad de Mexico 04510, Mexico; carcamo@unam.mx (A.M); 316298290@quimica.unam.mx (D.H.)
[3] Institute of Crystallography (IC), National Research Council (CNR), Via Salaria km 29,300, 00016 Rome, Italy; francesco.capitelli@ic.cnr.it
[4] Instituto de Ciencias Aplicadas y Tecnología, Universidad Nacional Autónoma de México, Circuito Exterior s/n, Cd. Universitaria, Ciudad de Mexico 045010, Mexico; selene.islas@icat.unam.mx
[5] Departamento de Química, Centro de Investigación y de Estudios Avanzados, Av. Instituto Politécnico Nacional 2508, Col. San Pedro Zacatenco, Ciudad de Mexico 07360, Mexico; mrosales@cinvestav.mx
[6] Department of Earth and Geoenvironmental Sciences, University of Bari "Aldo Moro", Via E. Orabona 4, 70125 Bari, Italy; michele.zema@uniba.it
[7] Institute of Crystallography (IC), National Research Council (CNR), Via Amendola 122/O, 70126 Bari, Italy
[8] Department of Chemistry, University of Pavia, Vialle Taramelli 16, 27100 Pavia, Italy; serenachiara.tarantino@unipv.it
[9] Institute of Geoscience and Georesources (IGG), National Research Council (CNR), Via Ferrata 1, 27100 Pavia, Italy
* Correspondence: dritan.siliqi@ic.cnr.it (D.S.); carcamo@unam.mx (A.M.)

Keywords: Membrane; Eggshell; Biomineralization; Intramineral proteins; Biocalcification; Biosilicification; Biomorphs.

Supplementary information includes the following information:

1. Identification of Intramineral Proteins by SDS-PAGE

Table S1. Fractions collected from injections made with 50 mM sodium citrate + 150 mM NaOH pH 4.0 at a flow rate of 0.5 mL/min. Fractions were collected every 1 mL and the volume in mL of the collected fractions is given. **Fxn:** Fraction.

**Fxn**	**1**	**2**	**3**	**4**	**5**	**6**
**Ostrich**	17.5	18	18.5	19	19.5	20
**Emu**	19	19.5	20	20.5	21	21.5

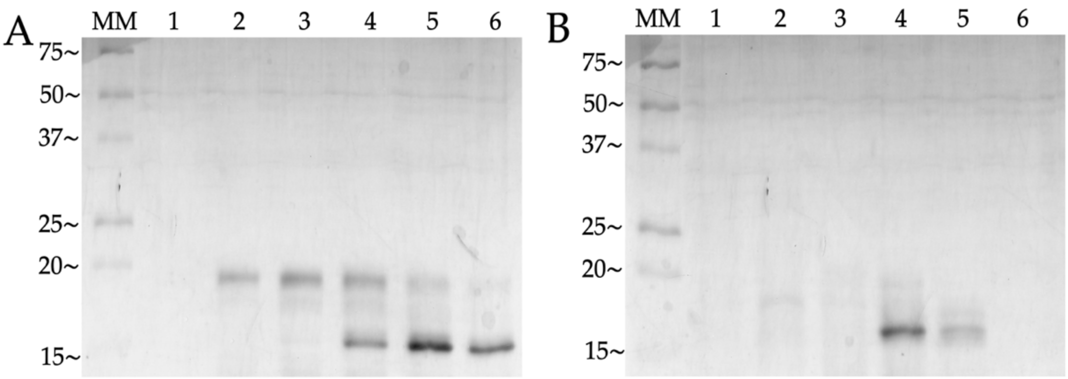

Figure S1. The fractions taken for the SDS-PAGE gel from ostrich (A) and emu (B) are listed in Table 1S with their respective volumes. The molecular weight marker (MM) corresponds to Prestained Protein Ladder (10 - 245 kDa).

Table S2. Fractions collected from injections made with 50 mM sodium citrate + 150 mM NaOH pH 4.0 at a flow rate of 0.5 mL/min. Fractions were collected every 1 mL and the volume in mL of the collected fractions is given. **Fxn:** Fraction.

**Fxn**	**1**	**2**	**3**	**4**	**5**	**6**	**7**	**8**
**C.** ***Acutus***	13	13.5	14	14.5	15	15.5	16	16.5
**C.** ***Moreletti***	13	13.5	14	14.5	15	15.5	16	--

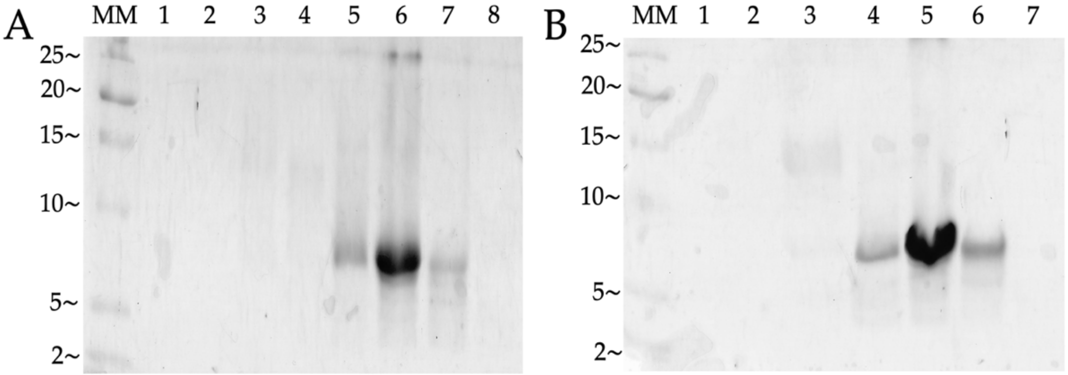

Figure S2. The fractions taken for the SDS-PAGE gel from crocodile *acutus* (A) and crocodile *moreletti* (B) are listed in Table 2S with their respective volumes. The molecular weight marker (MM) corresponds to Prestained Protein Ladder (10 - 245 kDa).

2. *Mass Spectrometry of Intramineral Proteins*

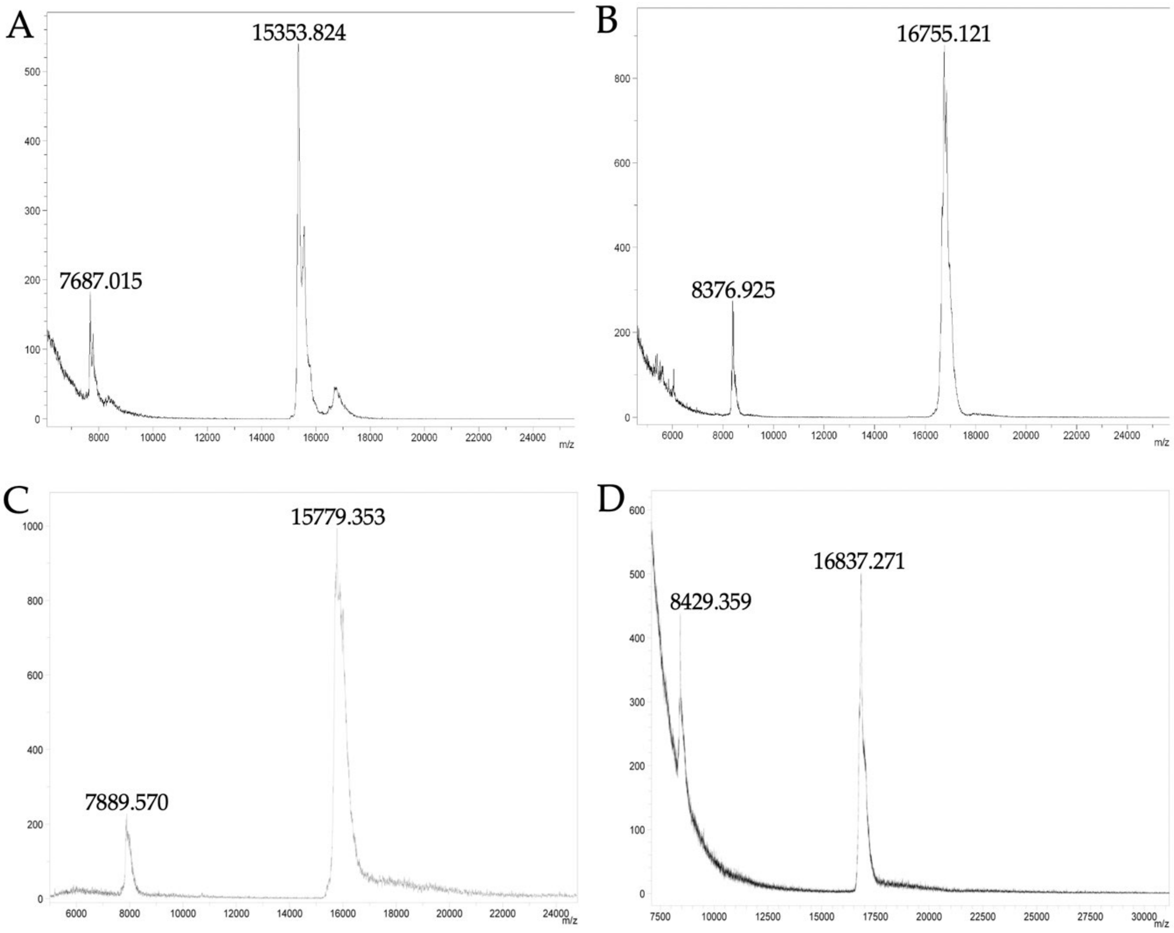

Figure S3. Mass spectra of intramineral proteins SCA-1 (A), SCA-2 (B), DCA-1 (C), and DCA-2 (D).

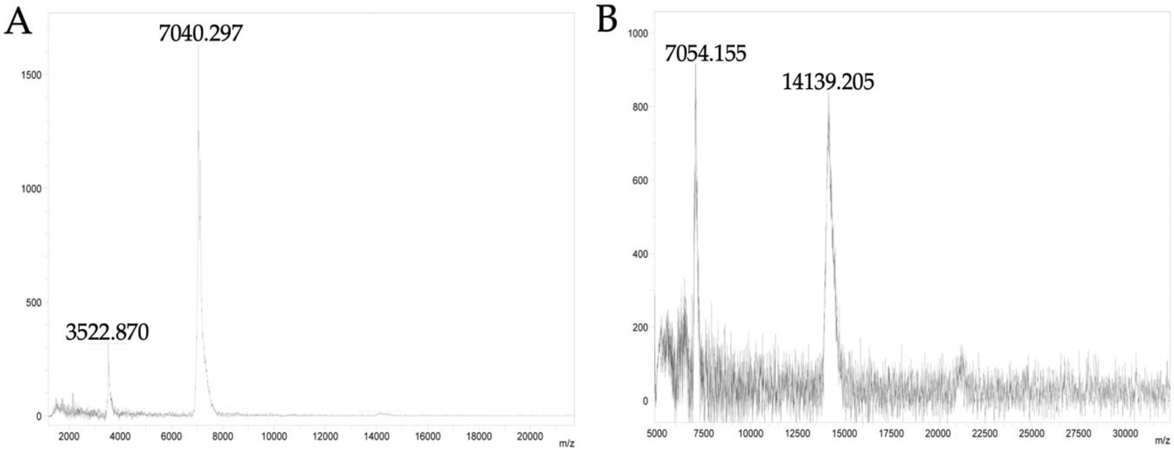

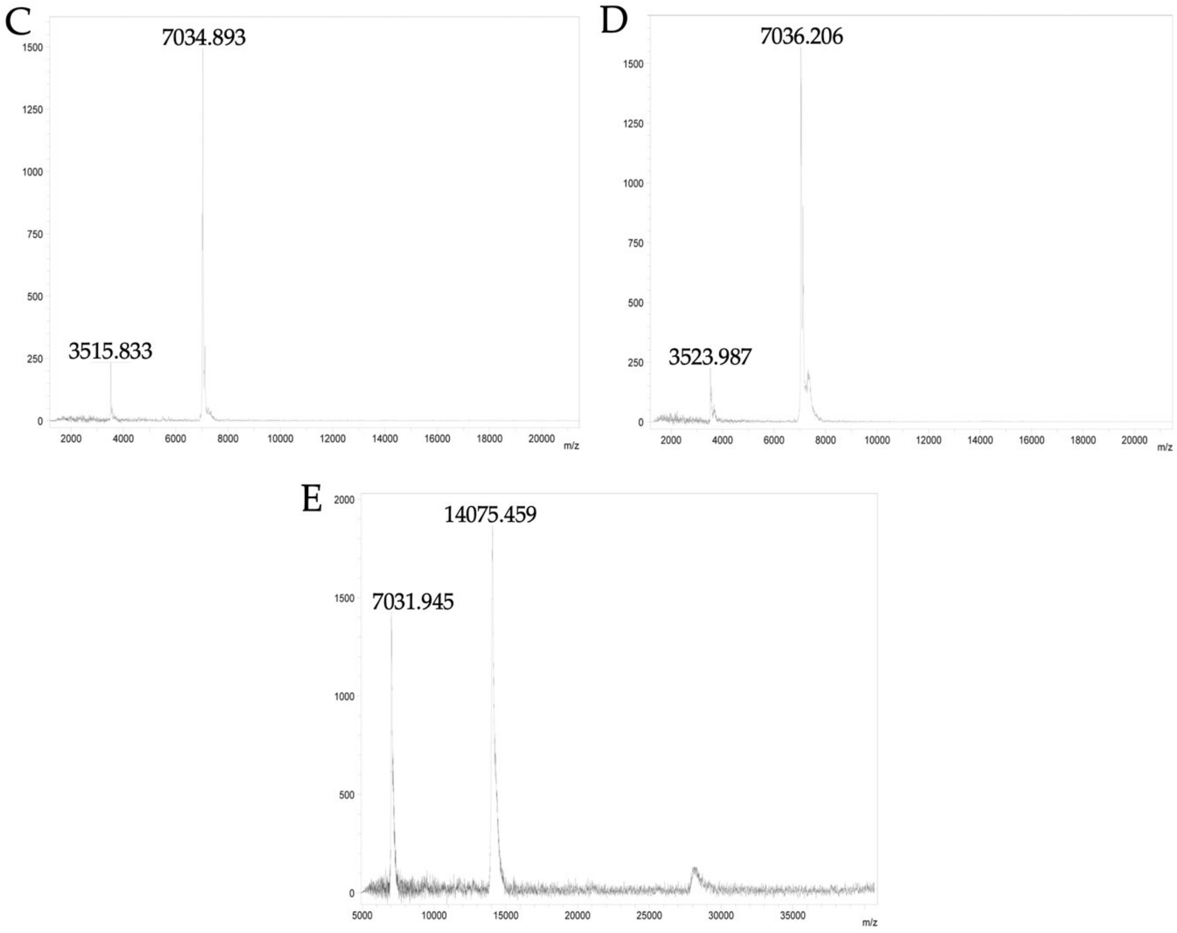

Figure S4. Mass spectra of intramineral proteins CCA-7 (A), CCA-14 (B), CCM-1 (C), CCM-2 (D), and CCM-3 (E).

3. Biomorphs Formation

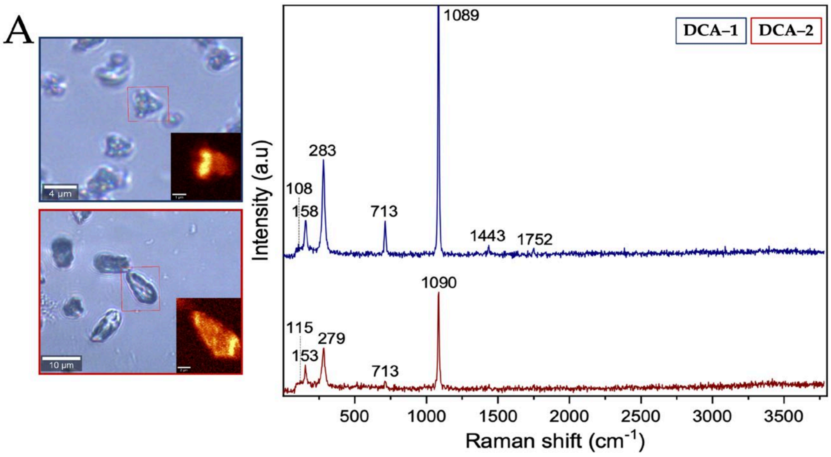

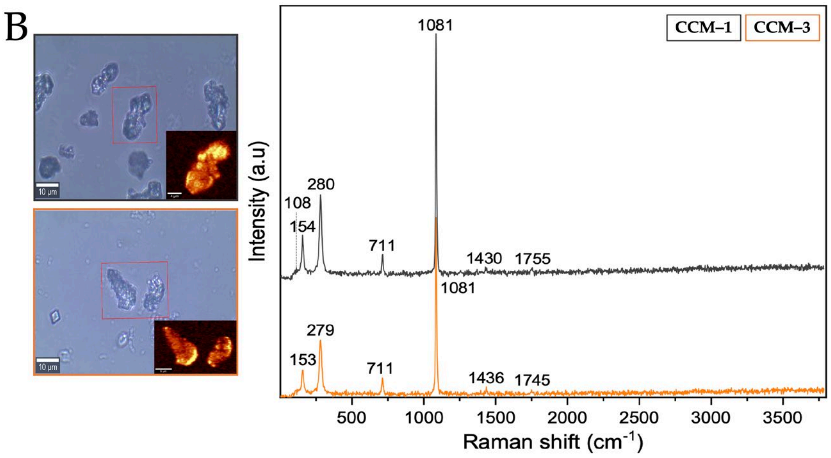

Figure S5. Raman spectra of calcium silica-carbonates synthesized. A: Dromaiocalcins; B: Crococalcins from crocodrile *moreletti*. The blue images are optical images, and the smaller ones correspond to the mapping performed on the biomorphs.

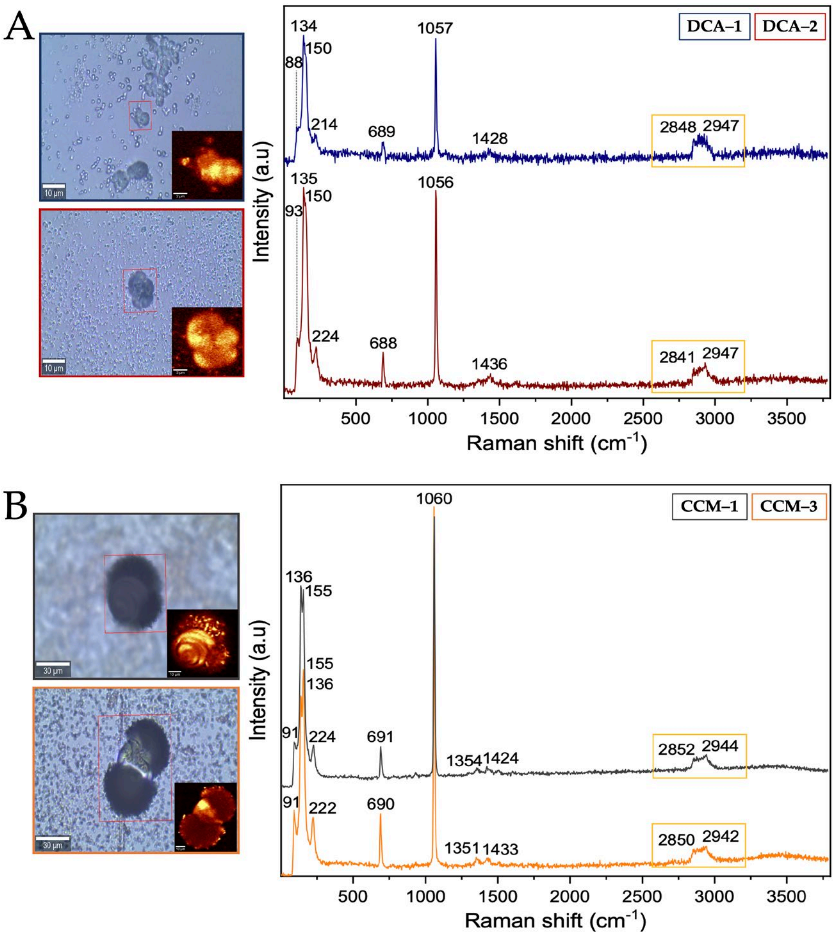

Figure S6. Raman spectra of barium silica-carbonates synthesized. A: Dromaiocalcins; B: Crococalcins from crocodrile *moreletti*. The blue images are optical images, and the smaller ones correspond to the mapping performed on the biomorphs. The yellow box indicates the protein signal.

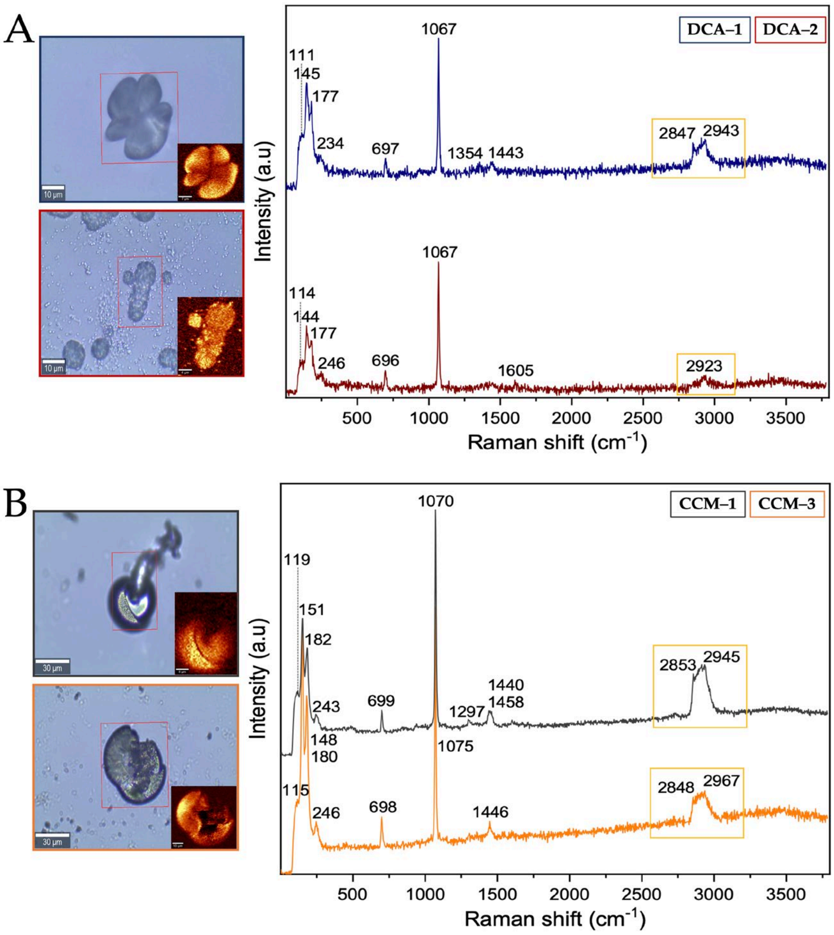

Figure S7. Raman spectra of strontium silica-carbonates synthesized. A: Dromaiocalcins; B: Crococalcins from crocodrile *moreletti*. The blue images are optical images, and the smaller ones correspond to the mapping performed on the biomorphs. The yellow box indicates the protein signal.

4. Elemental Analysis of the membranes of the ratite birds and reptiles eggshell

Table S3. Elemental percentage present in the membranes of the ratite birds and crocodiles eggshell.

Element	Ostrich	Emu	C. *Acutus*	C. *Moreletti*
**C**	55.97	55.86	55.01	59.50
**N**	11.56	15.47	17.18	11.80
**O**	25.30	24.26	23.86	24.39
**S**	4.03	3.70	3.95	4.31
**Na**	1.16	0.33	--	--
**Mg**	0.43	--	--	--
**Cl**	1.55	--	--	--
**Si**	--	0.38	--	--

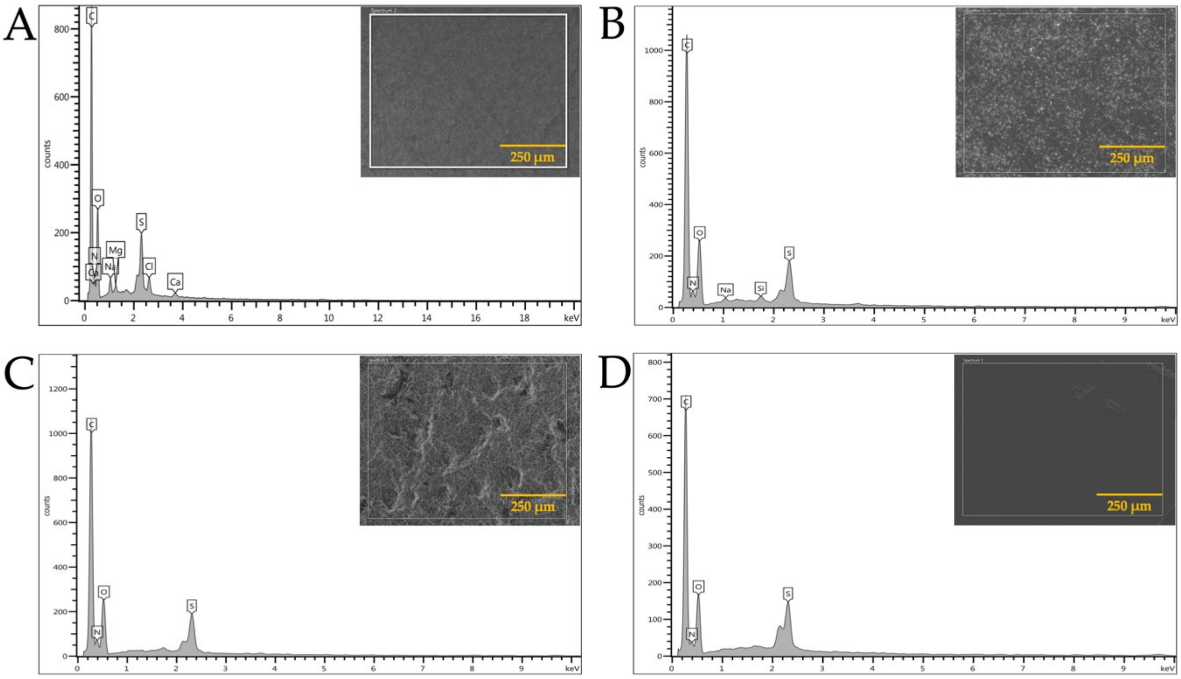

Figure S8. SEM-EDS images of the area outlined in white corresponding to the analysis performed on the eggshells membranes of ratite birds and crocodriles. A: Ostrich; B: Emu; C: C. *acutus*; D: C. *moreletti*.

5. Morphology of Eggshells of Birds and Crocodiles

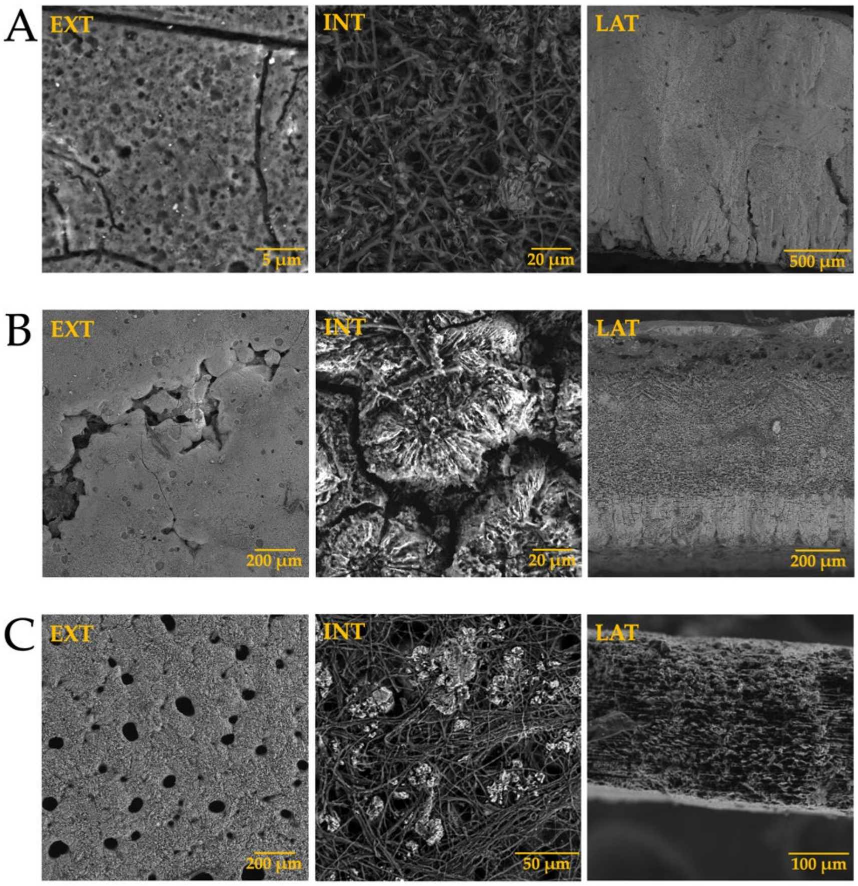

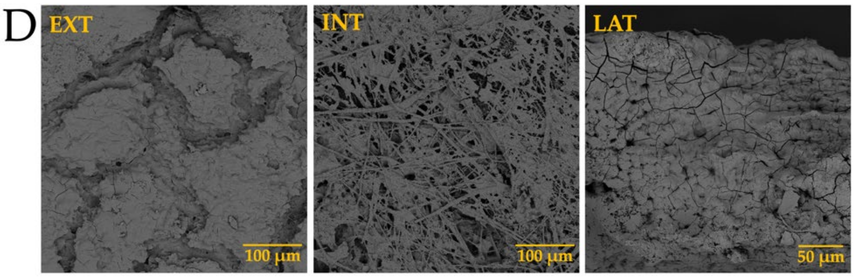

Figure S9. SEM images of ostrich (A), emu (B), crocodile *acutus* (C), and crocodile *moreletti* (D) eggshells. Ext: Outer part; Int: Inner part; Lat: Lateral/Intermediate section.

6. FTIR Analysis of Membranes of Birds and Crocodiles Eggshells

Table S4. Assignment of the FTIR vibrations of untreated membranes of ostrich before and after synthesis of calcium biomorphs. WO: Without $CaCO_3$; WT: With $CaCO_3$; SCA: Struthicalcin.*

Group	WO	WT	SCA-1	SCA-2
**Amide A**	3290	3284	3278	3278
**Amide B**	3068	3068	3068	3068
**νCH_2 ASYM**	2960	2960	2954	2960
**νCH_2 SYM**	2929	2929	2935	2929
**Amide I**	1635	1638	1635	1635
**Amide II**	1528	1509	1516	1516
**δCH_2**	1446	1446	1442	1442
**$\nu_3(CO_3)^{2-}$**	1398	1409	1405	1398
**Amide III**	1238	1238	1235	1235
**Blue Signal**	1072	1098	1079	1086
**$\nu_2(CO_3)^{2-}$**	--	872	872	872

* The shift is given in cm^{-1}.

Table S5. Assignment of the FTIR vibrations of untreated membranes of emu before and after synthesis of calcium biomorphs. WO: Without $CaCO_3$; WT: With $CaCO_3$; DCA: Dromaiocalcin.*

Group	WO	WT	DCA-1	DCA-2
**Amide A**	3278	3278	3274	3274
**Amide B**	3060	3060	3060	3060
**νCH_2 ASYM**	2960	2956	2960	2952
**νCH_2 SYM**	2926	2930	2926	2930
**Amide I**	1635	1632	1632	1632
**Amide II**	1528	1522	1522	1525
**δCH_2**	1448	1448	1448	1448
**$\nu_3(CO_3)^{2-}$**	1404	1398	1396	1396
**Amide III**	1234	1234	1234	1231
**Blue Signal**	1080	1080	1077	1077
**$\nu_2(CO_3)^{2-}$**	--	874	871	--

* The shift is given in cm^{-1}.

Table S6. Assignment of the FTIR vibrations of untreated membranes of crocodile *acutus* before and after synthesis of calcium biomorphs. WO: Without $CaCO_3$; WT: With $CaCO_3$; CCA: Crococalcin.*

Group	WO	WT	CCA-7	CCA-14
**Amide A**	3322	3271	3315	3278
**Amide B**	3081	--	--	3081
**$\nu CH_{2\ ASYM}$**	2966	--	--	2954
**$\nu CH_{2\ SYM}$**	2929	--	--	2929
**Amide I**	1635	1635	1635	1635
**Amide II**	1540	1521	1521	1514
**δCH_2**	1445	1432	1432	1438
**$\nu_3(CO_3)^{2-}$**	1400	1407	1394	1400
**Amide III**	1241	1216	1229	1229
**Blue Signal**	1071	1077	1077	1077
**$\nu_2(CO_3)^{2-}$**	--	874	874	874

* The shift is given in cm^{-1}.

Table S7. Assignment of the FTIR vibrations of untreated membranes of crocodile *moreletti* before and after synthesis of calcium biomorphs. WO: Without $CaCO_3$; WT: With $CaCO_3$; CCM: Crococalcin.*

Group	WO	WT	CCM-1	CCM-3
**Amide A**	3284	3278	3290	3278
**Amide B**	3081	3062	--	3068
**$\nu CH_{2\ ASYM}$**	2960	2960	--	2960
**$\nu CH_{2\ SYM}$**	2929	2922	--	2929
**Amide I**	1632	1635	1639	1632
**Amide II**	1517	1513	1517	1517
**δCH_2**	1444	1437	1434	1437
**$\nu_3(CO_3)^{2-}$**	1392	1399	1402	1409
**Amide III**	1236	1222	1218	1225
**Blue Signal**	1072	1072	1072	1076
**$\nu_2(CO_3)^{2-}$**	--	871	871	874

* The shift is given in cm^{-1}.

Table S8. Assignment of the FTIR vibrations of biocalcified membranes of ostrich before and after synthesis of calcium biomorphs. WO: Without $CaCO_3$; WT: With $CaCO_3$; SCA: Struthicalcin.*

Group	WO	WT	SCA-1	SCA-2
**$(OH)^-$**	3367	--	--	--
**Amide A**	3265	3284	3284	3284
**$\nu CH_{2\ ASYM}$**	2922	2966	2954	2960
**$\nu CH_{2\ SYM}$**	2852	2929	2922	2922
**Amide I**	1635	1641	1648	1641
**Amide II**	1540	1527	1521	1534

**δCH_2**	1457	1438	1451	1445
**$\nu_3(CO_3)^{2-}$**	1432	1407	1407	1407
**Amide III**	1248	1241	1241	1235
**$\nu_{1,3}(PO_4)^{3-}$**	1102<br>1026	1089<br>1019	1089<br>1026	1083<br>1019
**$\nu_2(CO_3)^{2-}$**	--	867	874	867
**$\nu_{2,4}(PO_4)^{3-}$**	664<br>550	601<br>557	601<br>557	601<br>557

* The shift is given in cm^{-1}.

Table S9. Assignment of the FTIR vibrations of biocalcified membranes of emu before and after synthesis of calcium biomorphs. WO: Without $CaCO_3$; WT: With $CaCO_3$; DCA: Dromaiocalcin.*

Group	WO	WT	DCA-1	DCA-2
**$(OH)^-$**	3341	--	--	--
**Amide A**	3284	3271	3278	3278
**νCH_2 ASYM**	2992	2966	2960	2960
**νCH_2 SYM**	2929	2929	2922	2929
**Amide I**	1635	1635	1635	1635
**Amide II**	1534	1527	1527	1521
**δCH_2**	1445	1438	1445	1438
**$\nu_3(CO_3)^{2-}$**	1413	1407	1407	1407
**Amide III**	1235	1235	1235	1235
**$\nu_{1,3}(PO_4)^{3-}$**	1096<br>1026	1096<br>1032	1089<br>1032	1089<br>1026
**$\nu_2(CO_3)^{2-}$**	--	867	867	874
**$\nu_{2,4}(PO_4)^{3-}$**	601<br>557	601<br>557	601<br>557	601<br>557

* The shift is given in cm^{-1}.

Table S10. Assignment of the FTIR vibrations of biocalcified membranes of crocodile *acutus* before and after synthesis of calcium biomorphs. WO: Without $CaCO_3$; WT: With $CaCO_3$; CCA: Crococalcin.*

Group	WO	WT	CCA-7	CCA-14
**$(OH)^-$**	3360	--	--	--
**Amide A**	3290	3278	3284	3296
**Amide I**	1641	1635	1648	1648
**Amide II**	1534	1514	1514	1521
**δCH_2**	1470	1445	1445	1451
**$\nu_3(CO_3)^{2-}$**	1420	1407	1407	1407
**Amide III**	1235	1235	--	--
**$\nu_{1,3}(PO_4)^{3-}$**	1083<br>1019	1077<br>1026	1089<br>1019	1089<br>1019
**$\nu_2(CO_3)^{2-}$**	--	874	867	867
**$\nu_{2,4}(PO_4)^{3-}$**	595	601	601	601

	557	550	557	557

* The shift is given in cm^{-1}.

Table S11. Assignment of the FTIR vibrations of biocalcified membranes of crocodile *moreletti* before and after synthesis of calcium biomorphs. WO: Without $CaCO_3$; WT: With $CaCO_3$; CCM: Crococalcin.*

Group	WO	WT	CCM-1	CCM-3
**$(OH)^-$**	3380	3385	3385	--
**Amide A**	3271	3278	3284	3278
**$\nu CH_{2\ ASYM}$**	2966	2966	2966	2966
**$\nu CH_{2\ SYM}$**	2935	2929	2935	2935
**Amide I**	1635	1635	1641	1635
**Amide II**	1534	1527	--	1514
**δCH_2**	1451	1451	1445	1445
**$\nu_3(CO_3)^{2-}$**	1413	1400	1407	1400
**Amide III**	1229	1235	1241	1235
**$\nu_{1,3}(PO_4)^{3-}$**	1071<br>1032	1077<br>1032	1077<br>1045	1077<br>1045
**$\nu_2(CO_3)^{2-}$**	--	--	874	874
**$\nu_4(PO_4)^{3-}$**				

* The shift is given in cm^{-1}.

Table S12. Assignment of the FTIR vibrations of biosilicified membranes of ostrich before and after synthesis of calcium biomorphs. WO: Without $CaCO_3$; WT: With $CaCO_3$; SCA: Struthiocalcin.*

Group	WO	WT	SCA-1	SCA-2
**$\nu(OH)^-$**	3373	--	--	--
**$\delta(OH)^-$**	1622	1635	1635	1641
**Amide III**	1172	1172	1179	1179
**$\nu(Si_2O)_{ASYM}$**	1071	1058	1052	1052
**$\nu(Si_2O)_{SYM}$**	956	944	950	956
**$\delta(Si_2O)$**	--	798	798	791

* The shift is given in cm^{-1}.

Table S13. Assignment of the FTIR vibrations of biosilicified membranes of emu before and after synthesis of calcium biomorphs. WO: Without $CaCO_3$; WT: With $CaCO_3$; SCA: Dromaiocalcin.*

Group	WO	WT	DCA-1	DCA-2
**$\nu(OH)^-$**	3385	--	--	--
**$\delta(OH)^-$**	1635	--	--	--
**Amide III**	1185	1172	1166	1172
**$\nu(Si_2O)_{ASYM}$**	1045	1052	1058	1058
**$\nu(Si_2O)_{SYM}$**	950	956	956	956
**$\delta(Si_2O)$**	791	798	791	791

* The shift is given in cm^{-1}.

Table S14. Assignment of the FTIR vibrations of biosilicified membranes of crocodile *acutus* before and after synthesis of calcium biomorphs. WO: Without $CaCO_3$; WT: With $CaCO_3$; CCA: Crococalcin.*

Group	WO	WT	CCA-7	CCA-14
**ν(OH)⁻**	3385	--	--	--
**δ(OH)⁻**	1635	1635	1635	1635
**Amide III**	1172	1166	1159	1159
**ν(Si_2O) ASYM**	1058	1039	1045	1039
**ν(Si_2O) SYM**	944	944	956	950
**δ(Si_2O)**	791	791	791	791

* The shift is given in cm^{-1}.

Table S15. Assignment of the FTIR vibrations of biosilicified membranes of crocodile *moreletti* before and after synthesis of calcium biomorphs. WO: Without $CaCO_3$; WT: With $CaCO_3$; CCA: Crococalcin.*

Group	WO	WT	CCM-1	CCM-3
**ν(OH)⁻**	3373	--	--	--
**δ(OH)⁻**	1628	1635	1635	1635
**Amide III**	1172	1166	1191	1185
**ν(Si_2O) ASYM**	1045	1052	1052	1045
**ν(Si_2O) SYM**	944	963	963	963
**δ(Si_2O)**	791	791	791	791

* The shift is given in cm^{-1}.
